# The Photoactive Photosynthetic Reaction Center of a Rhodobacter sphaeroides Mutant Lacking 3-Vinyl (Bacterio)Chlorophyllide *a* Hydratase Contains 3-Vinyl Bacteriochlorophyll *a*

**DOI:** 10.1128/spectrum.03878-22

**Published:** 2023-03-27

**Authors:** June Kim, Changwon Kim, Siin Kim, Hyotcherl Ihee, Woonsup Shin, Eui-Jin Kim, Jeong K. Lee

**Affiliations:** a Department of Life Science, Sogang University, Seoul, Republic of Korea; b Department of Chemistry and KI for the BioCentury, Korea Advanced Institute of Science and Technology (KAIST), Daejeon, Republic of Korea; c Center for Advanced Reaction Dynamics, Institute for Basic Science, Daejeon, Republic of Korea; d Department of Chemistry, Sogang University, Seoul, Republic of Korea; e Microbial Research Department, Nakdonggang National Institute of Biological Resources, Gyeongsangbuk-do, Republic of Korea; University of Minnesota Twin Cities

**Keywords:** *bchF* mutation, *Rhodobacter sphaeroides*, 3-vinyl bacteriochlorophyll *a*, reaction center

## Abstract

Rhodobacter sphaeroides mutant BF—lacking 3-vinyl (bacterio)chlorophyllide *a* hydratase (BchF)—accumulates chlorophyllide *a* (Chlide *a*) and 3-vinyl bacteriochlorophyllide *a* (3V-Bchlide *a*). BF synthesizes 3-vinyl bacteriochlorophyll *a* (3V-Bchl *a*) through prenylation of 3V-Bchlide *a* and assembles a novel reaction center (V-RC) using 3V-Bchl *a* and Mg-free 3-vinyl bacteriopheophytin *a* (3V-Bpheo *a*) at a molar ratio of 2:1. We aimed to verify whether a *bchF*-deleted R. sphaeroides mutant produces a photochemically active RC that facilitates photoheterotrophic growth. The mutant grew photoheterotrophically—implying a functional V-RC—as confirmed by the emergence of growth-competent suppressors of *bchC*-deleted mutant (BC) under irradiation. Suppressor mutations in BC were localized to *bchF*, which diminished BchF activity and caused 3V-Bchlide *a* accumulation. *bchF* expression carrying the suppressor mutations in *trans* resulted in the coproduction of V-RC and wild-type RC (WT-RC) in BF. The V-RC had a time constant (τ) for electron transfer from the primary electron donor P (a dimer of 3V-Bchl *a*) to the A-side containing 3V-Bpheo *a* (H_A_) similar to that of the WT-RC and a 60% higher τ for electron transfer from H_A_ to quinone A (Q_A_). Thus, the electron transfer from H_A_ to Q_A_ in the V-RC should be slower than that in the WT-RC. Furthermore, the midpoint redox potential of P/P^+^ of the V-RC was 33 mV more positive than that of the WT-RC. R. sphaeroides, thus, synthesizes the V-RC when 3V-Bchlide *a* accumulates. The V-RC can support photoheterotrophic growth; however, its photochemical activity is inferior to that of the WT-RC.

**IMPORTANCE** 3V-Bchlide *a* is an intermediate in the bacteriochlorophyll *a* (Bchl *a*)-specific biosynthetic branch and prenylated by bacteriochlorophyll synthase. R. sphaeroides synthesizes V-RC that absorbs light at short wavelengths. The V-RC was not previously discovered because 3V-Bchlide *a* does not accumulate during the growth of WT cells synthesizing Bchl *a*. The levels of reactive oxygen species increased with the onset of photoheterotrophic growth in BF, resulting in a long lag period. Although the inhibitor of BchF is unknown, the V-RC may act as a substitute for the WT-RC when BchF is completely inhibited. Alternatively, it may act synergistically with WT-RC at low levels of BchF activity. The V-RC may broaden the absorption spectra of R. sphaeroides and supplement its photosynthetic ability at various wavelengths of visible light to a greater extent than that by the WT-RC alone.

## INTRODUCTION

**FIG 1:**
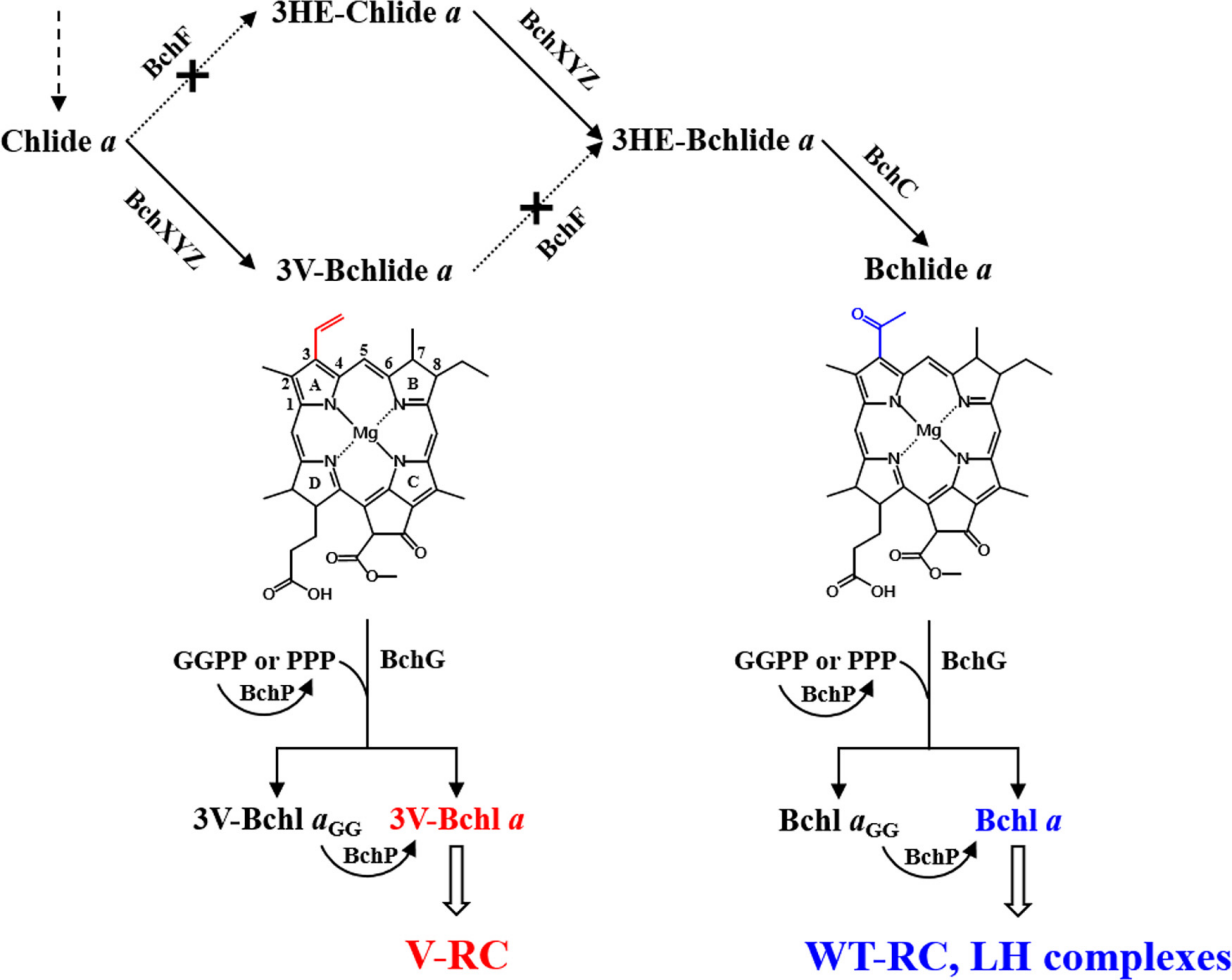
Biosynthesis of 3V-Bchl *a* in R. sphaeroides mutant BF lacking BchF. Bchl *a* is synthesized from Chlide *a* via BchF, BchXYZ, BchC, BchG, and BchP and used to form the wild-type reaction center (WT-RC) and light-harvesting (LH) complexes. BF (see Table S1 in the supplemental material) was constructed after deletion of *bchF* (black crosses), which resulted in cellular accumulation of Chlide *a* and 3V-Bchlide *a*. 3V-Bchlide *a* is phytylated by BchG and BchP, and the resulting 3V-Bchl *a* can form the novel RC (V-RC). Carbon numbering and pyrrole-ring designation are indicated with the structure of 3V-Bchlide *a*. Differences at C-3 between 3V-Bchlide *a* and Bchlide *a* are shown with red and blue lines, respectively. The metabolic pathways to form Bchlide *a* with 3HE-Chlide *a* and 3HE-Bchlide *a* are also illustrated.

Chlorophyllide *a* (Chlide *a*, Fig. 1) is phytylated to form chlorophyll *a* (Chl *a*) in oxygenic photosynthetic organisms or metabolized to bacteriochlorophyllide *a* (Bchlide *a*), which is then phytylated to produce bacteriochlorophyll *a* (Bchl *a*) in anoxygenic photosynthetic bacteria (Fig. 1). Bchlide *a* synthesis requires the reduction of a double bond between C-7 and C-8 of Chlide *a* by Chlide *a* reductase (COR; BchXYZ) to yield 3-vinyl Bchlide *a* (3V-Bchlide *a*), followed by hydration of the 3V group to the 3-hydroxyethyl (3HE) group of 3HE-Bchlide *a* by 3-vinyl (bacterio)chlorophyllide *a* [3V-(B)Chlide *a*] hydratase (BchF) (Fig. 1). Alternatively, Chlide *a* may be metabolized by BchF to yield 3HE-Chlide *a*, followed by reduction of the C-7–C-8 double bond by COR to form 3HE-Bchlide *a* (Fig. 1) and oxidation of the 3HE group by 3HE-Bchlide *a* dehydrogenase (BchC) to form the acetyl group of Bchlide *a*, which is a direct precursor of Bchl *a* (1). The metabolic intermediates of this Bchl *a*-specific biosynthetic branch are thought to be channeled through metabolic enzymes and do not accumulate in cells.

Deletion of *bchF* in Rhodobacter sphaeroides and Rhodobacter capsulatus results in the accumulation of Chlide *a* and 3V-Bchlide *a* (2–4). Phytylated 3V-Bchl *a* was found in the *bchF*-deleted mutant of R. sphaeroides, not Bchl *a* (5). These results indicate that Bchl *a* synthase (BchG) of R. sphaeroides can esterify accumulated 3V-Bchlide *a* with phytol (5). The physiological significance of 3V-Bchl *a* in the *bchF* mutant remains to be determined.

Bchl *a* of R. sphaeroides is integrated into light-harvesting (LH) complexes (LH1, LH2-1, and LH2-2 encoded by *pufBA*, *puc1BA*, and *puc2BA*, respectively) and a reaction center (RC) which consists of three subunits: L (*pufL*), M (*pufM*), and H (*puhA*) (6–8). The RC cofactors include four Bchl *a* molecules, two bacteriopheophytin *a* (Bpheo *a*; Bchl *a* without the central Mg^2+^) molecules, two ubiquinone (Q) molecules, a nonheme iron, and a carotenoid (9–11). They occur in symmetric pairs designated an A- and a B-branch, respectively. Despite the symmetry, electron transfer is thermodynamically feasible only in the A-branch (12). The special pair (P) of Bchl *a* is excited by light to form an excited state (P*) and undergoes charge separation, wherein an electron is emitted from P to form P^+^. One electron from P* is transferred to an accessory Bchl *a* (B_A_; subscript A denotes the A-branch) and subsequently to Bpheo *a* (H_A_). A tightly bound ubiquinone (Q_A_) accepts an electron from H_A_ and transfers it to a loosely bound ubiquinone (Q_B_), possibly through Fe^3+^ between the two Qs (13). The electron loss of P is recovered by cytochrome *c*_2_—a membrane-associated mobile electron carrier—to complete the turnover of electron transfer. Two turnovers convert Q_B_ to Q_B_H_2_ (quinol), which leaves the RC and delivers electrons to the cytochrome *bc*_1_ complex to generate a proton motive force for ATP synthesis (11).

We assessed whether Bchl *a* and Bpheo *a* (collectively called bacteriochlorin) in the RC of R. sphaeroides could be substituted with other chemically modified bacteriochlorins or Chl *a in vitro*. For example, Bpheo *a* at the H-site could be replaced by exogenous pheophytin *a* (Pheo *a*) when provided in excess amounts in the presence of mild detergent (such as lauryldimethylamine oxide [LDAO]) heated at 42°C (14–18). The RC modified with Pheo *a* exhibits electron transfer kinetics with a lower quantum yield for triplet P formation and Q reduction than that of the wild-type RC (WT-RC) (15, 18). Bchl *a* at the B-site cannot be replaced by a chlorin (Chl *a*, 13^2^-hydroxy Chl *a*, and 3-acetyl Chl *a*) (4). However, Bchl *a* at the B-site and Bpheo *a* at the H-site were successfully substituted with 3V-Bchl *a* and 3V-Bpheo *a*, respectively (4), but the resulting RC was not analyzed for electrochemical activity. Interestingly, a *bchD* (coding for magnesium chelatase)-deleted mutant of R. sphaeroides (19) formed Zn-Bchl *a* (harboring central Zn^2+^), which amounted to ~20% of the Bchl *a* of WT cells. The mutant cell produces a Zn-RC, wherein all six bacteriochlorins are substituted with Zn-Bchls *a* (20–22). The Zn-RC exhibits electron transfer and charge separation yields comparable to those of the WT-RC (22). Nonetheless, the *bchD* mutant was unable to grow photoheterotrophically (19, 23). Thus, the pigments of WT-RC could be replaced with other derivatives of Bchl *a* and Chl *a*. However, no pigment-modified RC enabled the cell to grow under photoheterotrophic condition. In this study, we examined whether the R. sphaeroides mutant BF lacking BchF (which accumulates 3V-Bchlide *a* and 3V-Bchl *a*) produces a photochemically active RC that facilitates photoheterotrophic growth.

## RESULTS

### R. sphaeroides mutant BF lacking BchF grows photoheterotrophically.

When Bchl *a* biosynthesis is blocked in R. sphaeroides, photosynthetic growth is assumed to be inhibited in the resulting mutant. Surprisingly, photoheterotrophically competent survivors of an R. sphaeroides mutant lacking BchC (BC; see Table S1 in the supplemental material) were obtained at a frequency of ~10^−8^ of inoculum cells in Sistrom’s succinate-based (Sis) minimal medium in an approximately 5-week incubation at 30°C, the suppressor mutations of which were mapped to *bchF* (see below). The result suggested that the R. sphaeroides mutant lacking BchF (BF; Table S1) can grow photoheterotrophically despite being unable to synthesize Bchl *a*.

**FIG 2:**
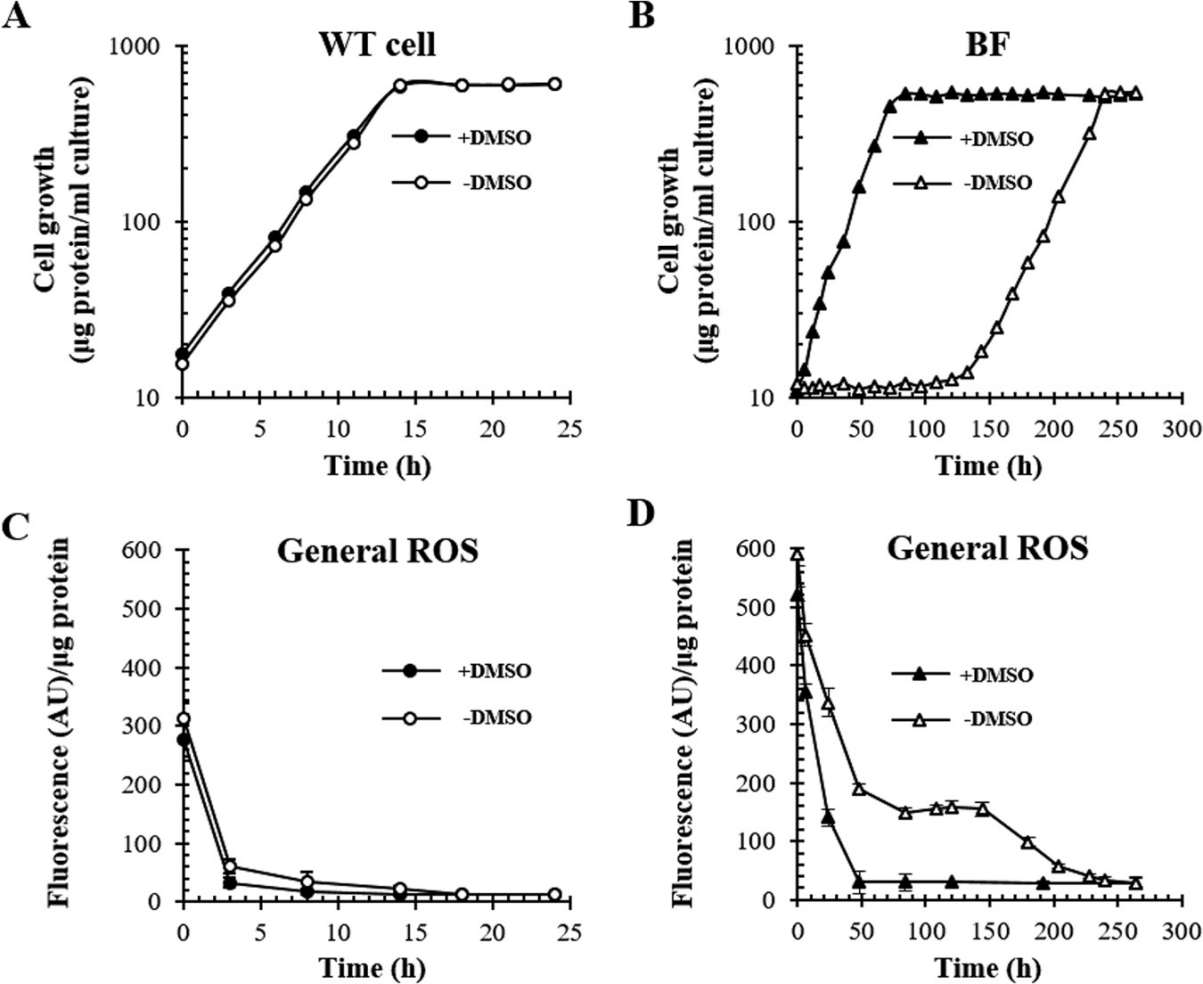
Effect of DMSO (10 mM) on photoheterotrophic growth of BF. (A and B) Photoheterotrophic growth of WT (A) and BF (B) cells at 15 W/m^2^ was recorded based on the total cellular protein in the presence (closed symbols) or absence (open symbols) of DMSO. (C and D) General ROS levels were determined using aliquots of exponentially growing cells with H_2_DCFDA as an indicator and normalized to cellular proteins. Each point represents the mean ± standard deviation (SD) from three independent experiments. Note that time scales of panels A and C are different from those of panels B and D.

Previously, it was shown that the accumulated 3V-Bchlide *a* is prenylated in an R. sphaeroides mutant lacking BchF (5) and that 3V-Bchl *a* and 3V-Bpheo *a* can occupy the B- and H-sites of the RC, respectively (4). Accordingly, we investigated whether BF can grow photosynthetically with RC consisting of 3V-Bch *a* and 3V-Bpheo *a*. The photoheterotrophic growth of BF in Sistrom’s succinate-based (Sis) minimal medium was examined based on cellular protein content instead of culture turbidity as the latter would be biased by the pigments accumulated by BF. No apparent growth was observed for approximately 130 h after the onset of photoheterotrophic culture, after which the mutant grew at 0.039 h^−1^ (doubling time of 17 h, without 10 mM dimethyl sulfoxide [DMSO]) (Fig. 2B). The lag period and growth rate were constant over three consecutive repetitions of aerobic cultivation with the photoheterotrophically grown cells after the lag; cells showing exponential growth were used as an inoculum for photoheterotrophic culture (data not shown). General reactive oxygen species (ROS) levels in BF during the lag were higher than those for photoheterotrophically growing WT cells and declined as BF grew (Fig. 2A to D).

**FIG 3:**
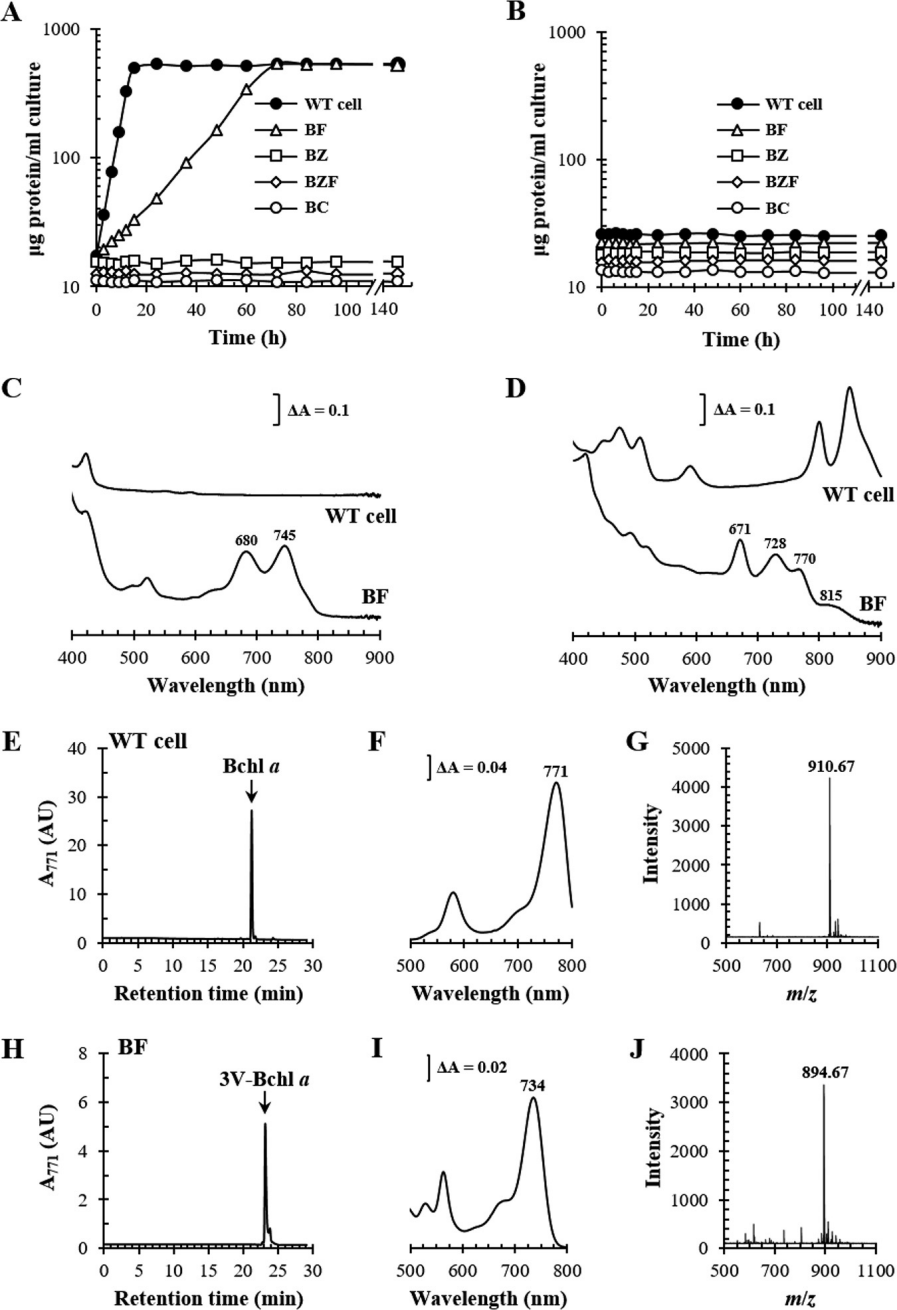
Photoheterotrophic growth and pigment analysis of mutants deficient in Bchl *a* synthesis genes. (A and B) WT, BF, BZ (Δ*bchZ*), BZF (Δ*bchZF*), and BC (Δ*bchC*) cells (see Table S1 in the supplemental material) were grown with (A) or without (B) light in the presence of 10 mM DMSO. Cell growth was estimated based on total cellular protein levels. (C) Absorption spectra of pigment accumulation in the culture supernatant. (D) Membranes were obtained and adjusted to equivalent protein levels. Absorption spectra of the membranes are illustrated with λ_max_ values indicated above each peak. (E to J) Phytylated pigments were extracted from the membranes of WT (E) and BF (H) cells and subjected to HPLC analysis at 771 nm. Pigments comprising the major peaks (black arrows) were pooled, and the absorption spectra (F and I) were recorded with the λ_max_ of the major Q_y_ bands. Pooled pigments were further subjected to cold-spray-TOF-MS analysis, and the masses of molecular ion (M^+·^) of the pigments from WT (G) and BF (J) cells were determined. AU, arbitrary units.

DMSO is a hydroxyl radical scavenger (24). ROS levels decreased in WT and BF cells following supplementation of the culture medium with 10 mM DMSO (Fig. 2C and D). BF immediately grew in the presence of DMSO (0.051 h^−1^, with a doubling time of 13.8 h) (Fig. 2B), which was ~22% of the growth rate of WT cells (0.231 h^−1^, with a doubling time of 3.0 h) (Fig. 2A). The photoheterotrophic growth of BF (Fig. 2B) cannot be attributed to anaerobic respiration since 10 mM DMSO did not promote the growth of BF and WT cells in the dark under anaerobic conditions (Fig. 3B). The lag period of BF growth was consistently shortened by the additional expression of *sodA* (encoding Mn-superoxide dismutase [MnSOD]) (Fig. S1A and F) or *sodB* (encoding Fe-superoxide dismutase [FeSOD]) (Fig. S1B and F) of Vibrio vulnificus, and their coexpression led to a much shorter lag period (Fig. S1C and F). However, heterologous expression of V. vulnificus catalase (KatG) did not affect the lag period (Fig. S1D and G). Thus, superoxide and hydroxyl radical may be generated after the onset of photoheterotrophic growth in BF and could be responsible for the lag period. Taken together, BF appears competent for growth under photoheterotrophic conditions with the lag prolonged by general ROS levels.

Other mutants lacking Bchl *a*-specific biosynthetic enzymes were examined for photoheterotrophic growth in a medium supplemented with 10 mM DMSO (Fig. 3A). No growth was observed with the mutants BZ (lacking the BchZ subunit of COR), BZF (lacking both COR and BchF), and BC (lacking BchC) (Fig. 1 and 3A and Table S1), which was consistent with the results of previous studies (3, 23). The biosynthetic intermediates accumulated by the mutants under dark anaerobic conditions were as follows: Chlide *a* and 3V-Bchlide *a* by BF, 3HE-Chlide *a* by BZ, Chlide *a* by BZF, and 3HE-Chlide *a* and 3HE-Bchlide *a* by BC (Fig. S2). Since BF grows photoheterotrophically, it is evident that 3V-Bchlide *a* (λ_max_ at 745 nm) (Fig. 3C), not Chlide *a* (λ_max_ at 680 nm) (Fig. 3C), would be used in the formation of photosynthetic complexes.

Unlike the WT cell membrane, the spectrum of the BF membrane presented peaks at 815, 770, 728, and 671 nm (Fig. 3D). Total pigments were extracted from the BF membrane by liquid-liquid phase extraction using an acetone-methanol (7:2, vol/vol) mixture and *n*-hexane. Phytylated pigments move to the upper organic *n*-hexane layer, whereas pigments without phytyl groups are localized in the lower aqueous acetone layer (25). The pigment(s) in the aqueous layer contained Chlide *a* and 3V-Bchlide *a* according to high-performance liquid chromatography (HPLC) (Fig. S2). Meanwhile, the major pigment in the organic layer was 3V-Bchl *a* (Fig. 3H) with a λ_max_ at 734 nm (Fig. 3I) and a molecular mass of 894.67 (Fig. 3J) according to HPLC and cold-spray-time of flight mass spectrometry (TOF-MS). Bchl *a* (Fig. 3E) with a λ_max_ at 771 nm (Fig. 3F) and a molecular mass of 910.67 (Fig. 3G) was observed in the membranes of WT cells. Bpheo *a* and 3V-Bpheo *a* were also detected in WT (Fig. S3A and C) and BF (Fig. S3D and F) membranes, following Bchl *a* (Fig. S3A and B) and 3V-Bchl *a* (Fig. S3D and E) at absorbance wavelengths of 749 nm and 719 nm, respectively. Chlide *a* and 3V-Bchlide *a* from the BF membrane were blue-shifted to 671 nm and 728 nm, respectively (Fig. 3D), compared with those in the culture supernatant (Fig. 3C), presumably owing to the presence of a new photosynthetic complex including 3V-Bchl *a*, which exhibits spectral peaks at 815 nm and 770 nm and other peaks overlapping those of Chlide *a* and 3V-Bchlide *a* (Fig. 3D).

### 3V-Bchlide *a* was phytylated by BchG to form 3V-Bchl *a*.

The biosynthesis of 3V-Bchl *a* in *bchF*-deleted mutants (5, 26) by BchG is not kinetically characterized. BchG functions via a ping-pong mechanism with geranylgeranyl pyrophosphate (GGPP) or phytyl pyrophosphate (PPP) as the first substrate and Bchlide *a* as the second substrate (27). The C terminus of BchG was translationally fused to a His_6_ tag and overexpressed in Escherichia coli. The *k*_cat_ was determined by quantifying the amount of BchG in E. coli lysates by Western immunoblot analysis using an anti-His_6_ tag antibody with similarly sized C-terminally His_6_-tagged protoporphyrin ferrochelatase (PpfC-His_6_) of Vibrio vulnificus as the quantification standard (Fig. S4A and B) (28). 3V-Bchlide *a* was known to be unstable and easily demetallized to 3-vinyl bacteriopheophorbide *a* (3V-Bpheide *a*) (26). However, 3V-Bchlide *a* (λ_max_ at 734 nm)—distinguished from 3V-Bpheide *a* (λ_max_ at 719 nm)—remains stable (Fig. S5A).

**FIG 4:**
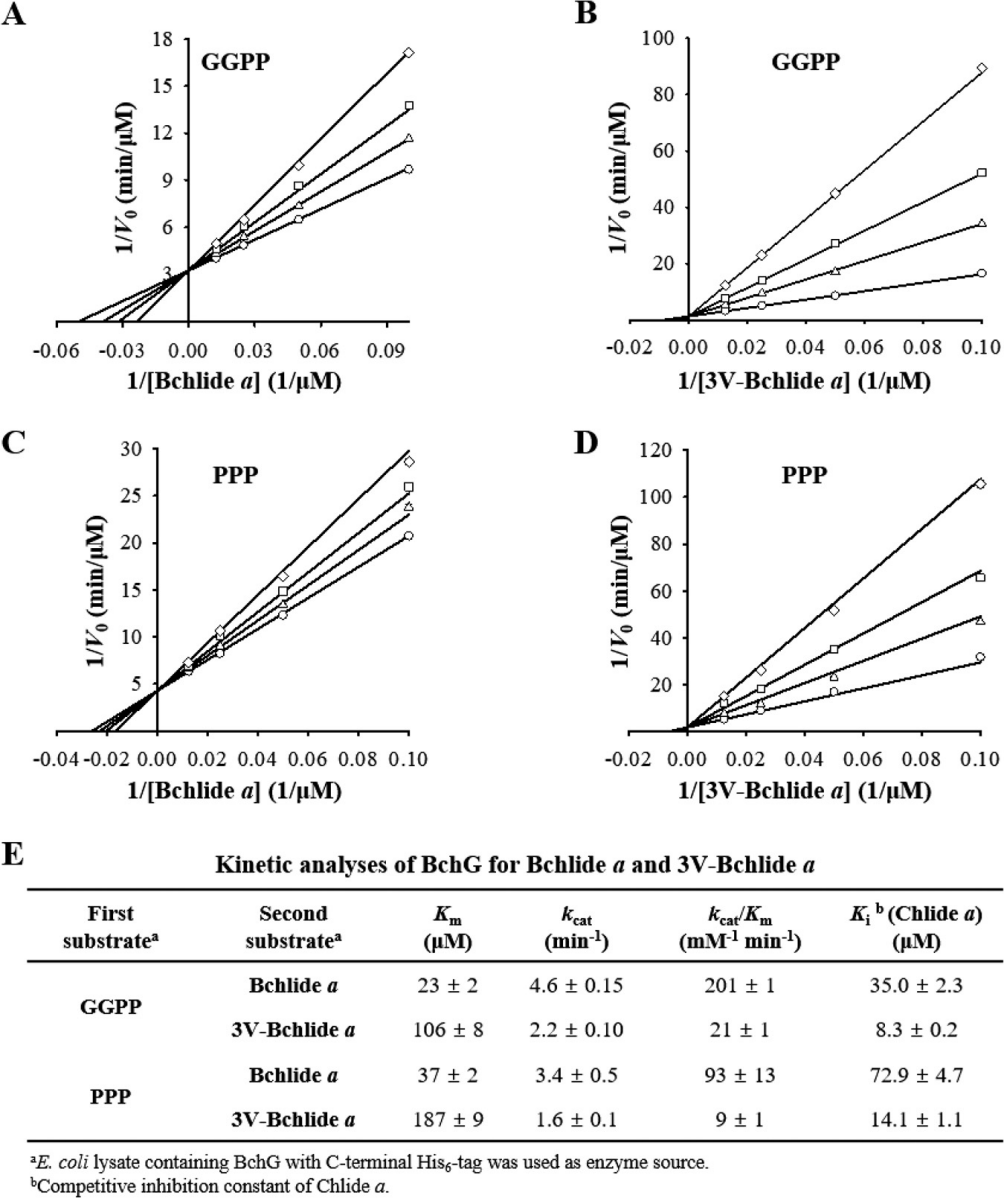
Kinetic analysis of BchG for 3V-Bchlide *a* and inhibition of BchG by Chlide *a*. Kinetic parameters (*K_m_* and *k*_cat_) of BchG for 3V-Bchlide *a* as the second substrate were determined using geranylgeranyl pyrophosphate (GGPP) or phytyl pyrophosphate (PPP) as the first substrate. Kinetic analysis for Bchlide *a* was performed for comparison. (A to D) Inhibition of BchG by Chlide *a* (circle, 0; triangle, 10 μM; square, 20 μM; diamond, 40 μM) was determined in the presence of Bchlide *a* (A and C) and 3V-Bchlide *a* (B and D). GGPP (A and B) or PPP (C and D) was included at 50 μM. (E) Data sets of *V*_0_ in double reciprocal plots were fitted to a competitive inhibition model using nonlinear regression to calculate the kinetic parameters. Each point was determined using three independent experiments.

BchG showed higher *K_m_* and lower *k*_cat_ values for 3V-Bchlide *a* than for Bchlide *a*, resulting in ~10-fold lower catalytic efficiency (*k*_cat_/*K_m_*, Fig. 4E). Chlide *a* accumulated in BF and is a competitive inhibitor of BchG (27). Competitive inhibition of BchG activity by Chlide *a* was four to five times stronger when 3V-Bchlide *a* was prenylated than when Bchlide *a* was used as a substrate based on the inhibition constant (*K_i_*) (Fig. 4). Thus, 3V-Bchlide *a* prenylation may be limited by the low catalytic efficiency of BchG and strong inhibition by Chlide *a*. Nonetheless, 3V-Bchl *a* is formed in BF, indicating that both limitations were overcome by the formation of a photosynthetic complex consisting of 3V-Bchl *a*. Given catalytic efficiencies, BchG would preferentially form Bchl *a* when both Bchlide *a* and 3V-Bchlide *a* are present at similar levels.

Geranylgeranyl reductase (BchP) catalyzes the reduction of GGPP and geranylgeranylated Bchl *a* (Bchl *a*_GG_) to PPP and Bchl *a*, respectively (Fig. 1). Geranylgeranylated 3V-Bchl *a* (3V-Bchl *a*_GG_) was not detected in the BF membrane (Fig. 3H); therefore, BchG prenylates 3V-Bchlide *a* with PPP but may first use GGPP to form 3V-Bchl *a*_GG_ (Fig. 4E), followed by BchP-catalyzed reductions of its geranylgeranyl group to form 3V-Bchl *a*.

### 3V-Bchl *a* was used to form the RC (V-RC) of BF.

**FIG 5:**
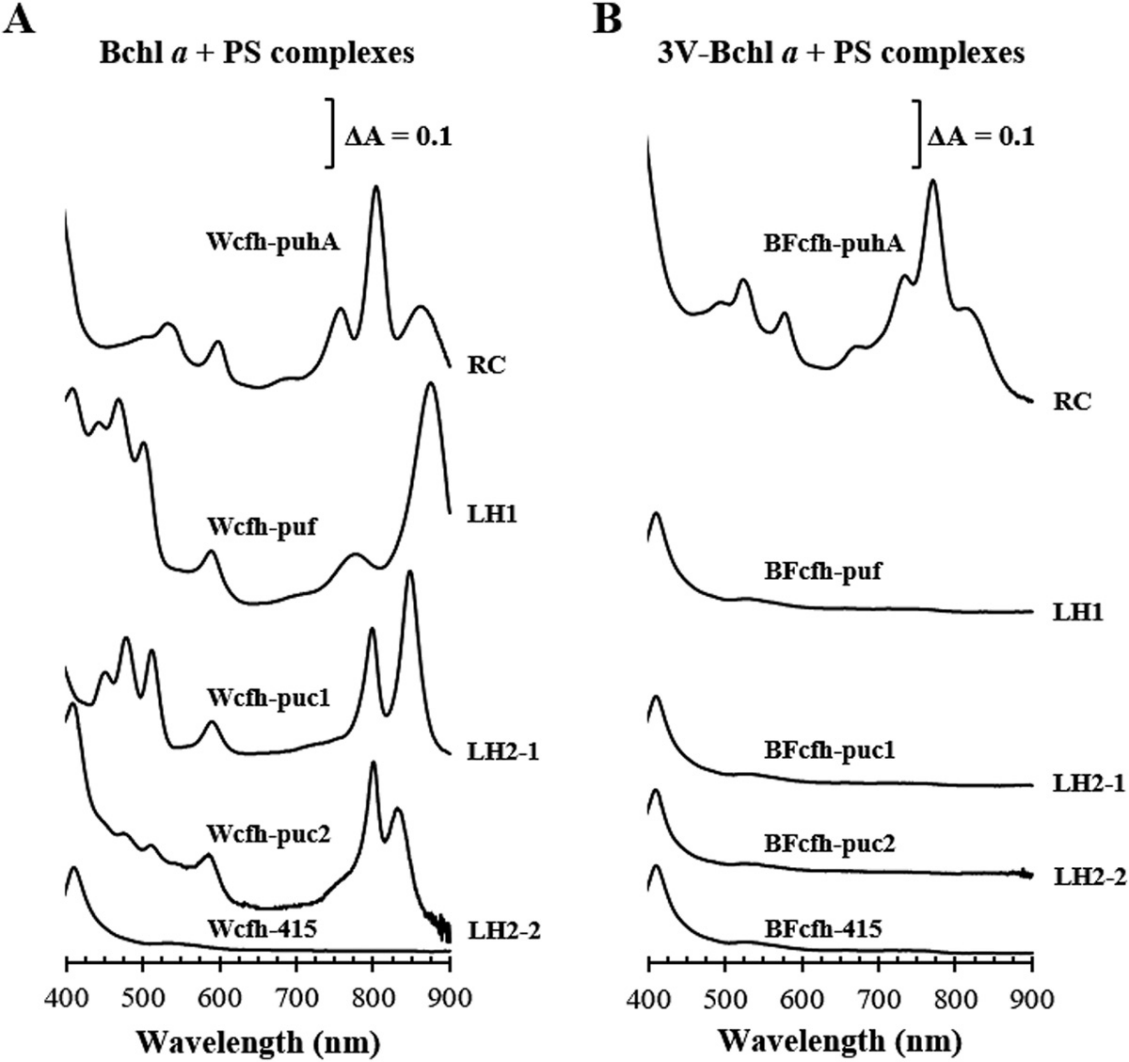
Photosynthetic complex formation using Bchl *a* and 3V-Bchl *a*. (A) Semiaerobically grown Wcfh-puhA, Wcfh-puf, Wcfh-puc1, and Wcfh-puc2 were used as controls for the formation of RC, LH1, LH2-1, and LH2-2, respectively. (B) Semiaerobically grown BFcfh-puhA, BFcfh-puf, BFcfh-puc1, and BFcfh-puc2 were used to examine RC, LH1, LH2-1, and LH2-2 formation, respectively, using 3V-Bchl *a*. Wcfh-415 and BFcfh-415 contain empty vectors. The photosynthetic complexes were purified by His tag affinity chromatography, and the absorption spectra are illustrated.

Formation of the photosynthetic complex with 3V-Bchl *a* was examined according to the expression of genes coding for the structural polypeptides of RC, LH1, LH2-1, and LH2-2 (Fig. 5B). Formation of the photosynthetic complex with Bchl *a* was examined as a control (Fig. 5A). Mutants of the BF cell and WT cell were generated: BFcfh (BF with *puc12BA*, *pufBA*, and *puhA* deletions) and Wcfh (WT with *puc12BA*, *pufBA*, and *puhA* deletions) (Table S1). The plasmid carrying *puhA* (the H subunit of RC) was mobilized in *trans* in BFcfh and Wcfh to yield BFcfh-puhA and Wcfh-puhA, respectively (Table S1). Similarly, recombinant strains were generated by expressing *pufBA* (BFcfh-puf and Wcfh-puf), *puc1BA* (BFcfh-puc1 and Wcfh-puc1), and *puc2BA* (BFcfh-puc2 and Wcfh-puc2) (Table S1). The C termini of PuhA, PufA, Puc1A, and Puc2A were fused to His_6_ tag in the plasmid constructs. Recombinant strains were grown semiaerobically, and each photosynthetic complex was purified by His tag affinity chromatography (Fig. 5). The RC, LH1, LH2-1, and LH2-2 were identified in Wcfh (Fig. 5A), whereas only the RC was observed in BFcfh-puhA (Fig. 5B). Thus, 3V-Bchl *a* was involved in the formation of only the RC, not the LH complexes (Fig. 5B).

3V-Bchl *a* was not detected in BFcfh-415 grown semiaerobically, but Chlide *a* and 3V-Bchlide *a* were detected at similar levels (Fig. S5B). Similar results were observed for BFcfh-puf, BFcfh-puc1, and BFcfh-puc2, wherein Chlide *a* and 3V-Bchlide *a* were found at levels similar to those in BFcfh-415 (Fig. S5B). Meanwhile, Chlide *a* and 3V-Bchlide *a* levels in BFcfh-puhA were 7- to 8-fold higher than those in BFcfh-415 (Fig. S5B). This demonstrated increased metabolic flow to 3V-Bchlide *a*.

### V-RC is photochemically active but inferior to WT-RC.

**FIG 6:**
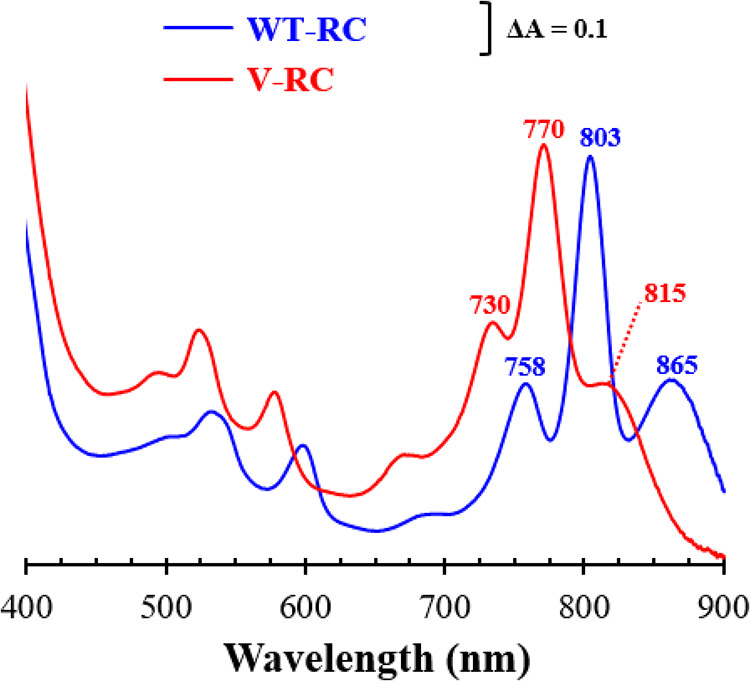
Ground-state absorption spectra of RCs. The WT-RC and V-RC were purified by His tag affinity chromatography from semiaerobically grown Wcfh-puhA and BFcfh-puhA, respectively. Ground-state absorption spectra were measured at 298 K. The Q_y_ regions are illustrated with the λ_max_ of the peaks. *A*_770_ of the V-RC was normalized to *A*_803_ of the WT-RC.

WT-RC and V-RC were purified by His tag affinity chromatography from the membranes of Wcfh-puhA and BFcfh-puhA grown under semiaerobic conditions. The ground-state absorption spectrum of the V-RC was blue-shifted in the Q_x_ (500- to 600-nm) bands and Q_y_ (700- to 900-nm) bands compared to those of the WT-RC (Fig. 6). Absorption peaks at 865, 803, and 758 nm of the WT-RC originate from the Q_y_ region of the P (Bchl *a*)-, B (Bchl *a*)-, and H (Bpheo *a*)-sites, respectively (29). We hypothesized that the V-RC peaks at 815, 770, and 730 nm correspond to P-, B-, and H-sites, respectively. All three peaks were blue-shifted, implying that the pigments at the P-, B-, and H-sites of the V-RC were different from those of the WT-RC. Thus, the 815-nm and 770-nm peaks observed in the membrane of photoheterotrophically cultured BF (Fig. 3D) were annotated as the Q_y_ peaks of the V-RC.

**FIG 7:**
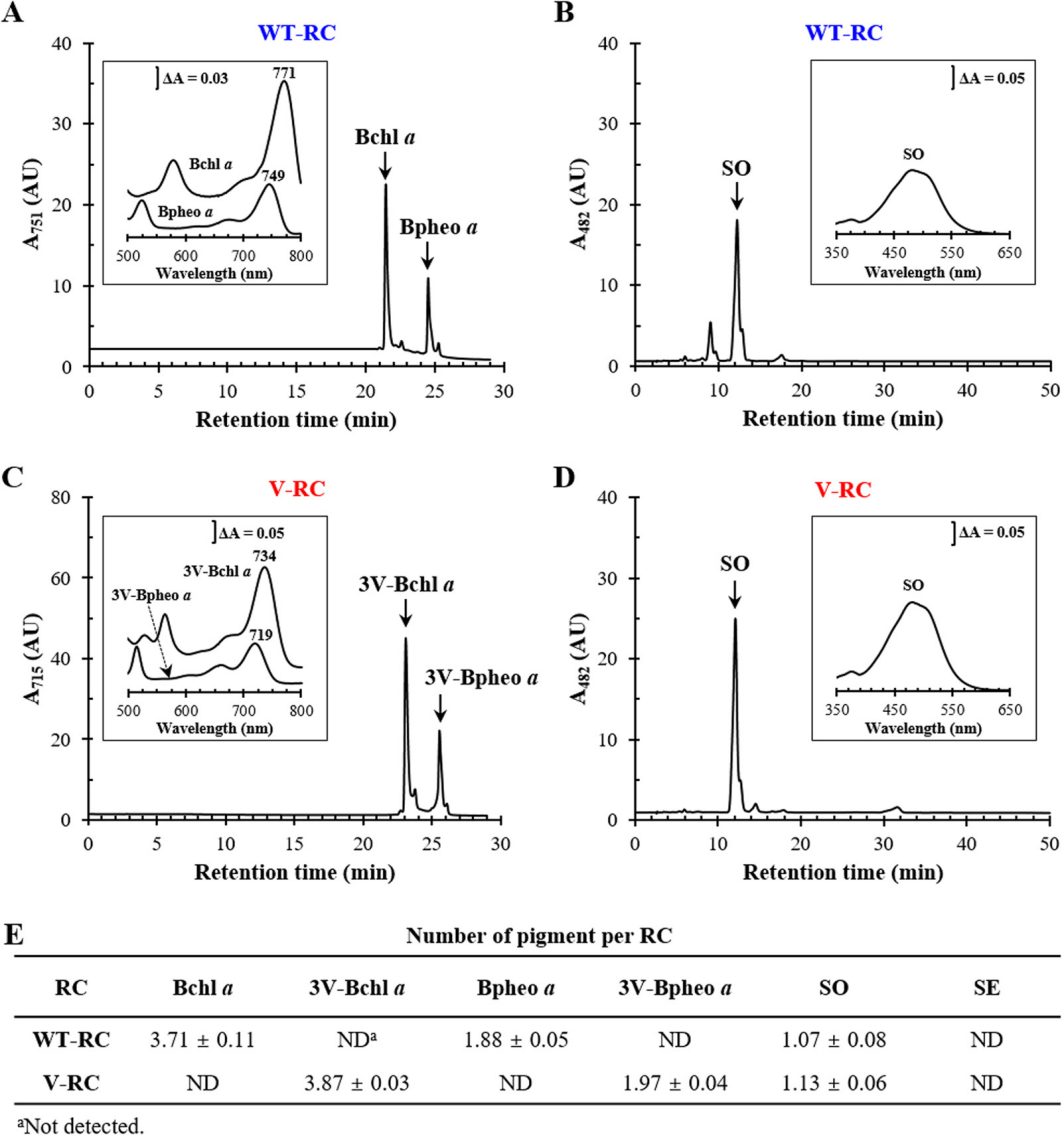
Pigment compositions of WT-RC and V-RC. (A to D) Pigments were extracted from WT-RC (A and B) and V-RC (C and D) produced from semiaerobically grown Wcfh-puhA and BFcfh-puhA, respectively. Bchl *a* (with Bpheo *a*) of the WT-RC (A), 3V-Bchl *a* (with 3V-Bpheo *a*) of the V-RC (C), and their carotenoids (B and D) were quantified using HPLC. (A) Bchl *a* and Bpheo *a* of the WT-RC were monitored at 751 nm (the two pigments had the same molar extinction coefficient). (C) 3V-Bchl *a* and 3V-Bpheo *a* of the V-RC were detected at 715 nm. (B and D) The carotenoid spheroidenone (SO) was monitored at 482 nm. The absorption spectrum of each pigment is illustrated with the λ_max_ of the prominent peaks in the insets of panels A to D. (E) The amounts of RCs were calculated using Western immunoblot analysis (see Fig. S4C and D in the supplemental material). The pigment content per RC is shown as the mean ± standard deviation. AU, arbitrary unit; SE, spheroidene.

The V-RC was digested with trypsin and α-chymotrypsin, and pigments were extracted with *n*-hexane and analyzed by HPLC (Fig. 7); the WT-RC was included as a control. The C terminus of PuhA was translationally fused to the His_6_ tag, and the RCs in the membranes of Wcfh-puhA and BFcfh-puhA were determined by Western immunoblot analysis using an anti-His_6_ tag antibody and the V. vulnificus PpfC-His_6_ (Fig. S4C and D) (28). The WT-RC contained approximately four Bchl *a* molecules, two Bpheo *a* molecules, and one spheroidenone (SO) molecule (Fig. 7A, B, and E), which correlated with the results of previous studies (9–11). The V-RC was estimated to contain four 3V-Bchl *a* molecules, two 3V-Bpheo *a* molecules, and one SO molecule (Fig. 7C, D, and E). Thus, Bchl *a* and Bpheo *a* of the WT-RC were replaced by 3V-Bchl *a* and 3V-Bpheo *a*, respectively, in the V-RC.

The V-RC was isolated from the membranes of photoheterotrophically grown BFcfh-puhA and analyzed in a manner similar to that for semiaerobically grown cells, with the WT-RC used as a control (Fig. S6A to C). The composition of cofactors of the V-RC (Fig. S6F, G, and H) and WT-RC (Fig. S6D, E, and H) was similar to that in semiaerobically grown cells (Fig. 7E), except for the presence of spheroidene (SE), which reflects the culture conditions. SO is formed by the enzymatic oxidation of SE by SE monooxygenase (CrtA) (30), and the ratio of SE to SO in photosynthetic complexes of R. sphaeroides is determined by oxygen availability. SO is dominant over SE under semiaerobic conditions and vice versa under photoheterotrophic conditions (30). The combined level of SO and SE of the V-RC from photoheterotrophically grown cells amounted to approximately one per RC complex (Fig. S6H), which was similar to that in the WT-RC of Wcfh-puhA (Fig. S6H).

BFcfh-puhA was cultured photoheterotrophically, and the total RC level in the cell membrane was quantified by Western immunoblot analysis of PuhA-His_6_ using an anti-His_6_ antibody and V. vulnificus PpfC-His_6_ (in the same manner as in Fig. S6B and C) (28). The total amounts of 3V-Bchl *a* and 3V-Bpheo *a* were also determined from the BFcfh-puhA membrane by HPLC. A comparison of the levels of phytylated pigments to those of RCs showed that most 3V-Bchl *a* and 3V-Bpheo *a* molecules were associated with RCs (Fig. S6I). Free phytylated pigments were almost absent in the membrane, and the level of RCs could be predicted from the amount of 3V-Bchl *a*.

**FIG 8:**
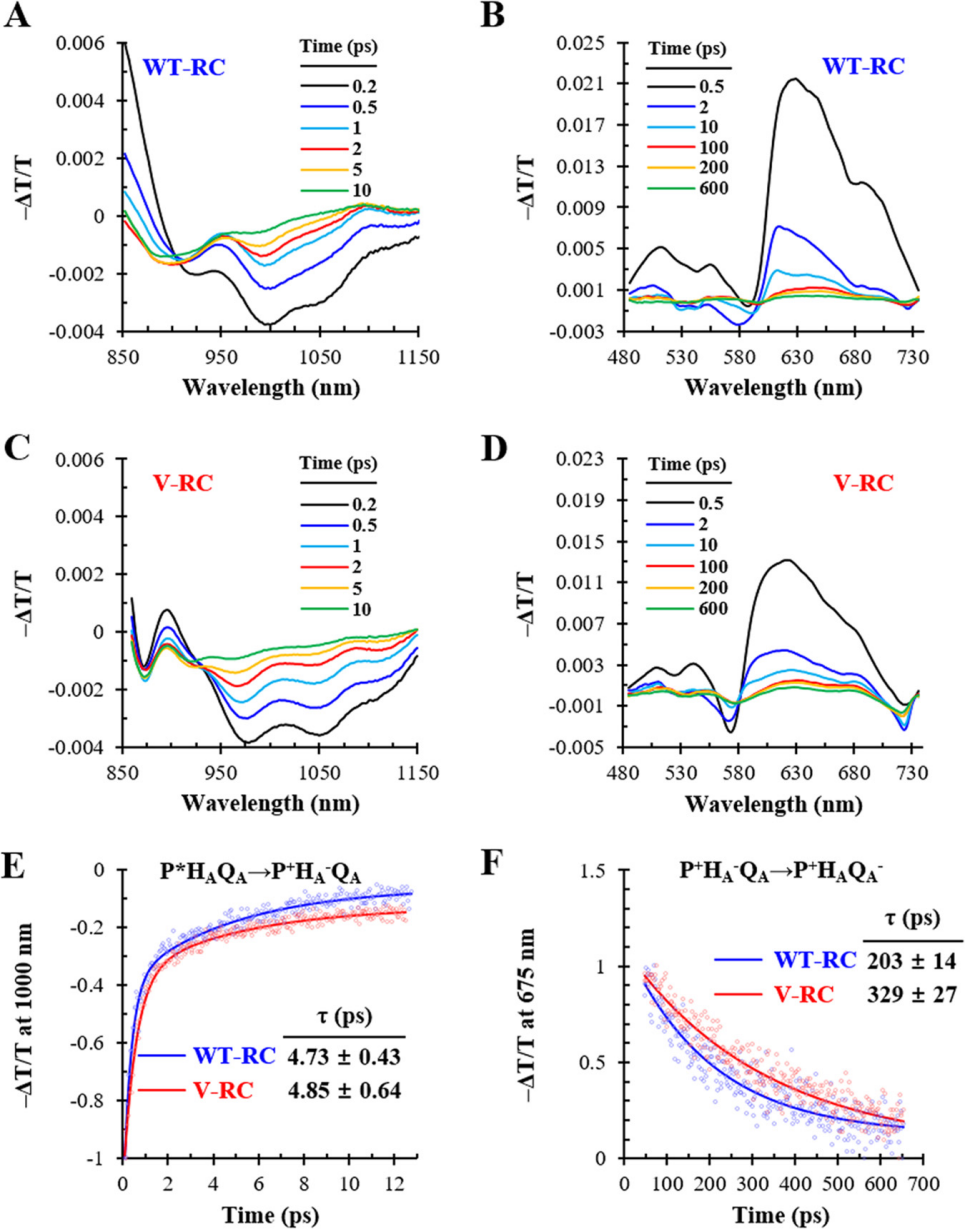
Broadband transient absorption spectroscopy of WT-RC and V-RC after photooxidation. (A to D) The WT-RC (A and B) and V-RC (C and D) were excited by an 800-nm laser, and the changes in absorption spectra were recorded in the near-infrared (A and C) and visible wavelength (B and D) regions in process of time. The absorbance changes at 1,000 nm and 675 nm were assigned to reflect the dynamics of the P*H_A_Q_A_ state and P^+^H_A−_Q_A_ state, respectively, in both RCs if the electron transfers follow the sequential transfer model. (E and F) Absorbance changes at 1,000 nm (E) and 675 nm (F) were tracked over time, where open circles and solid lines represent raw data points and fitting curves, respectively. The fitted time constants (τ) for P*H_A_Q_A_→P^+^H_A−_Q_A_ (E) and P^+^H_A−_Q_A_→P^+^H_A_Q_A−_ (F) are represented with fitting errors. T, transmittance.

The initial electron transfer kinetics of the V-RC were analyzed using broadband transient absorption spectroscopy to examine the coordinated effect of 3V-Bchl *a* and 3V-Bpheo *a* on the function of the RC (Fig. 8). Electrons are transferred via pigments upon light irradiation; therefore, the electronic states are presented in the specific absorption spectra. Excitation of the WT-RC by an 800-nm laser led to a rapid absorbance decrease in the 950- to 1,100-nm region at 0.2 ps (formation of P*), which corresponds to the stimulated emission signal of P* (Fig. 8A) (31). The dip gradually recovered within 10 ps, which presents the state change from P*H_A_Q_A_ to P^+^H_A−_Q_A_ (P→H_A_ electron transfer) (31). Meanwhile, an increase in absorbance occurred in the 600- to 700-nm region (formation of H_A−_) and gradually recovered within 600 ps (Fig. 8B), indicating a state change from P^+^H_A−_Q_A_ to P^+^H_A_Q_A−_ (H_A_→Q_A_ electron transfer) (32).

Excitation of the V-RC by an 800-nm laser resulted in similar spectral features, but the overall spectral regions were blue-shifted compared with those of the WT-RC (Fig. 8C and D). When applied to a sequential transfer model, the number of decaying components of the V-RC was the same as that of the WT-RC. Furthermore, their time scales were in ranges similar to the corresponding ones of the WT-RC. Consequently, the V-RC was assumed to have similar electron transfer kinetics. The time constant scales on the order of a few picoseconds and a few hundred picoseconds obtained from the absorbance changes at 1,000 nm and 675 nm were assigned to reflect the dynamics of P*H_A_Q_A_→P^+^H_A−_Q_A_ and P^+^H_A−_Q_A_→P^+^H_A_Q_A−_, respectively, in both RCs.

The absorbance changes of the WT-RC and V-RC were tracked at 1,000 nm (Fig. 8E) and 675 nm (Fig. 8F) after excitation, and τ values were obtained from fitting of the kinetic traces. The τ values for P*H_A_Q_A_→P^+^H_A−_Q_A_ and P^+^H_A−_Q_A_→P^+^H_A_Q_A−_ in the WT-RC were 4.73 ± 0.43 (Fig. 8E) and 203 ± 14 ps (Fig. 8F), respectively, which are similar to previous findings (22). Meanwhile, the τ value for P*H_A_Q_A_→P^+^H_A−_Q_A_ was 4.85 ± 0.64 ps (Fig. 8E) and the τ value for P^+^H_A−_Q_A_→P^+^H_A_Q_A−_ was 329 ± 27 ps (Fig. 8F) in the V-RC, the latter of which is 1.6-fold longer than that in the WT-RC. Thus, the electron transfer rate from P to H_A_ was comparable between RCs, whereas that from H_A_ to Q_A_ was slower in the V-RC than in the WT-RC.

**FIG 9:**
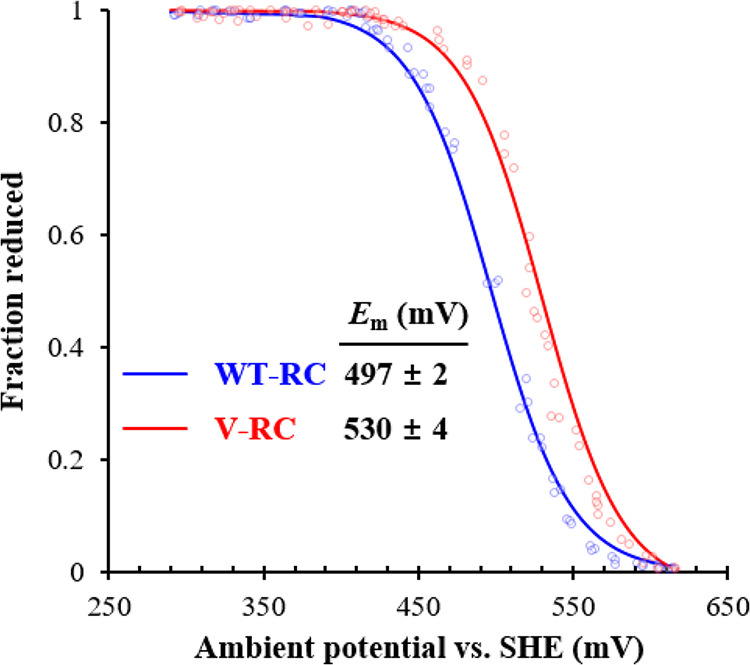
Electrochemical titration of P/P^+^ redox pairs in WT-RC and V-RC. WT-RC and V-RC were oxidatively titrated with potassium permanganate in the presence of potassium ferrocyanide as a mediator. The data from three independent titrations (open circles) were fitted (lines) to the Nernst equation with *n *= 1 (the number of electrons transferred in redox pair). The midpoint potentials (*E_m_*) of P/P^+^ for the WT-RC and V-RC were determined and presented as mean ± standard deviation. Ambient potentials were presented as the values versus the standard hydrogen electrode (SHE).

The redox potential (*E_m_*) of P/P^+^ is a crucial determinant of RC functionality. The *E_m_* values of P/P^+^ in the WT-RC and V-RC were determined via electrochemical titration using a ferro-ferricyanide system (Fig. 9). The *E_m_* of P/P^+^ in the WT-RC was 497 ± 2 mV, which is similar to that in a previous study (33). Meanwhile, the *E_m_* of P/P^+^ in the V-RC was 530 ± 4 mV (Fig. 9), which is more positive than that of the WT-RC by 33 mV. The similar τ values for P*H_A_Q_A_→P^+^H_A−_Q_A_ in both RCs indicated that the redox difference between P and H_A_ was comparable between the V-RC and WT-RC. Furthermore, we assumed no change in the redox of Q_A_ by the coordination of the RC with 3V derivatives of Bch *a* and Bpheo *a*. Accordingly, the redox difference between H_A_ and Q_A_ of the V-RC is thought to be lower than that of the WT-RC, which may extend the τ value for P^+^H_A−_Q_A_→P^+^H_A_Q_A−_ in the V-RC. Taken together, R. sphaeroides synthesizes functional V-RC with photochemical activity inferior to that of WT-RC.

### R. sphaeroides mutant BC yielded suppressor mutants growing photoheterotrophically; the second mutations were mapped to *bchF*.

BC did not grow photoheterotrophically (Fig. 3A). As mentioned earlier, however, suppressor colonies of BC appeared under photoheterotrophic condition after approximately 5 weeks in Sis minimal medium without DMSO or approximately 2 weeks in the same medium supplemented with DMSO (10 mM) after the photoheterotrophic culture with a frequency of ~10^−8^ of the cells in the inoculum. Three separate suppressor strains of BC (BCS1 to BCS3) (Table S1) grew photoheterotrophically (Fig. S7A). Interestingly, the absorption spectra of BCS cell lysates grown photoheterotrophically were similar to that of BF grown under the same conditions (Fig. S7B). The three BCSs and the parental BC were cultured under dark anaerobic conditions with 75 mM DMSO for comparative pigment analysis. The absorption spectra of the BCS cell lysates differed from that of BC (Fig. S7C) but were similar to that of BF (Fig. S7C). BCSs primarily accumulated Chlide *a* and 3V-Bchlide *a* and minor amounts of 3HE-Chlide *a* and 3HE-Bchlide *a* (Fig. S7D and E). These results implied that *bchF* is the second mutation in the BCSs. Sequence analysis of BCS1, BCS2, and BCS3 genomic DNA revealed point mutations leading to changes in residues at the 67th (Leu→Pro; L67P), 138th (Tyr→His; Y138H), and 101st (Asp→Asn; D101N) codon of BchF, respectively (Table S1).

Three BchF variants (BchF^L67P^, BchF^Y138H^, and BchF^D101N^) were expressed in E. coli, and cell lysates were used as enzyme sources for kinetic analysis (Table 1), with WT BchF used as a control. The amounts of BchF and its variants were quantified by Western immunoblot analysis with an anti-His_6_ tag antibody, and the similarly sized C-terminally His_6_-tagged carbonic anhydrase of R. sphaeroides (RsCA-His_6_ from E. coli BL-RsCA [Table S1]) was used as a quantification standard (Fig. S4E and F). All variants showed higher *K_m_* and lower *k*_cat_ values for Chlide *a* and 3V-Bchlide *a* than did the WT enzyme. The catalytic efficiencies (*k*_cat_/*K_m_*) of BchF^L67P^, BchF^Y138H^, and BchF^D101N^ for Chlide *a* (and 3V-Bchlide *a*) decreased to 7.5% (3.8%), 3.4% (2.5%), and 0.5% (0.5%), respectively, of the corresponding values of the WT enzyme. Thus, suppressor mutations of BCSs diminished BchF activities, resulting in cellular accumulation of Chlide *a* and 3V-Bchlide *a*, as observed with BF (Fig. S7D and E). These results further confirm that BF can grow photoheterotrophically, which was not revealed in WT cells owing to the lack of 3V-Bchlide *a* accumulation.

**TABLE 1:** Kinetic analyses of WT BchF and BchF variants for Chlide *a* and 3V-Bchlide *a*

Enzyme^*a*^	Substrate	*K_m_* (μM)	*k*_cat_ (min^−1^)	*k*_cat_/*K_m_* (mM^−1^ min^−1^)
BchF	Chlide *a*	151 ± 20	23.33 ± 1.28	154.48 ± 8.46
	3V-Bchlide *a*	186 ± 9	22.23 ± 0.25	119.46 ± 1.32
BchF^L67P^	Chlide *a*	214 ± 19	2.47 ± 0.10	11.52 ± 0.48
	3V-Bchlide *a*	396 ± 27	1.96 ± 0.16	4.59 ± 0.43
BchF^Y138H^	Chlide *a*	282 ± 23	1.50 ± 0.07	5.32 ± 0.23
	3V-Bchlide *a*	402 ± 33	1.21 ± 0.06	3.01 ± 0.14
BchF^D101N^	Chlide *a*	771 ± 73	0.58 ± 0.04	0.75 ± 0.05
	3V-Bchlide *a*	948 ± 55	0.56 ± 0.14	0.60 ± 0.15

a E. coli lysates containing BchFs with a C-terminal His_6_ tag were used as enzyme sources.

BCS phenotypes were reaffirmed by transforming mutant BCF cells lacking both BchC and BchF with plasmids carrying *bchF^L67P^*, *bchF^Y138H^*, and *bchF^D101N^* genes to yield BCF-L67P, BCF-Y138H, and BCF-D101N, respectively (Table S1). Controls included BCF recombinant strains with an empty plasmid (BCF-415) and a plasmid carrying WT *bchF* DNA (BCF-WT) (Table S1). All three BCF recombinant strains grew photoheterotrophically, similar to the control strain BCF-415, while BCF-WT showed no growth (Fig. S8A), which was similar to that of BC (Fig. 3A and Fig. S7A). The absorption spectra of the three BCF recombinant cell lysates were similar to that of BCF-415 (Fig. S8B), implying low BchF activities owing to the point mutations. Comparative pigment analysis involved growing all BCF recombinant strains under dark anaerobic conditions in 75 mM DMSO. The absorption spectra of the three BCF recombinant cell lysates varied from that of BCF-WT but were similar to those of BCF-415 (Fig. S8C) and BF (Fig. S7C). All three BCF recombinant strains primarily accumulated Chlide *a*, 3V-Bchlide *a*, and minor amounts of 3HE-Chlide *a* and 3HE-Bchlide *a* (Fig. S8D and E). Thus, BCF-L67P, BCF-Y138H, and BCF-D101N showed pigment accumulation similar to that of the corresponding suppressor strains BCS1, BCS2, and BCS3, respectively (Fig. S7D and E). Thus, BCSs gained the ability to grow photoheterotrophically through mutations that diminished BchF activity of BC.

### V-RCs can be formed together with WT-RCs when BchF activity is decreased.

**FIG 10:**
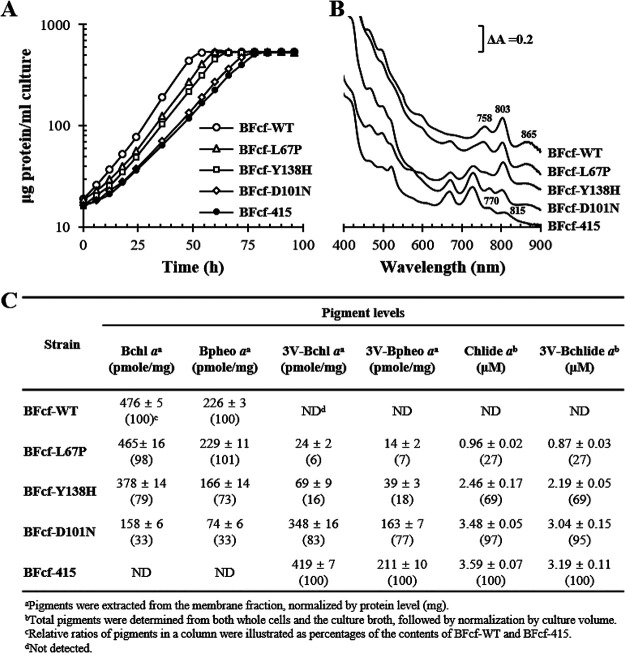
Characterization of RC-only strains producing Bchl *a*, 3V-Bchl *a*, or both Bchl *a* and 3V-Bchl *a*. Recombinant BFcf-WT (BFcf expressing *bchF*), BFcf-L67P (BFcf expressing *bchF^L67P^*), BFcf-Y138H (BFcf expressing *bchF^Y138H^*), BFcf-D101N (BFcf expressing *bchF^D101N^*), and BFcf-415 (BFcf carrying empty vector) were cultured photoheterotrophically in the presence of 10 mM DMSO. (A) Growth was determined based on the total protein content of the cells. (B) The membrane fractions were isolated and analyzed for absorption spectra with λ_max_ values shown on the peaks. (C) Pigment analysis in the membrane fractions. Chlide *a* and 3V-Bchlide *a* were extracted from whole cells and the culture broth, and each pigment was spectrally quantified.

The V-RC was blue-shifted compared to the WT-RC; therefore, the range of light absorption can be expanded if both RCs are formed in R. sphaeroides. Mutant BchFs were used instead of WT BchF for the biosynthesis of Bchl *a* as mutant BchFs have low catalytic efficiencies (Table 1) corresponding to reduced metabolic flow at the step mediated by the enzyme to accumulate 3V-Bchlide *a* while simultaneously synthesizing Bchl *a*. Plasmids containing *bchF^L67P^*, *bchF^Y138H^*, and *bchF^D101N^* (Table S1) were expressed in BFcf to yield BFcf-L67P, BFcf-Y138H, and BFcf-D101N, respectively (Table S1). The recombinant strains were grown photoheterotrophically (Fig. 10), and BFcf-415 and BFcf-WT (Table S1), which produce V-RC and WT-RC, respectively, were used as controls. BFcf-WT (growth rate of 0.071 h^−1^, doubling time of 9.8 h) showed a 1.5-fold-higher growth rate than BFcf-415 (growth rate of 0.052 h^−1^, doubling time of 14.2 h). The growth rate of the three recombinant strains (BFcf-L67P, BFcf-Y138H, and BFcf-D101N) was proportional to the catalytic efficiency of BchF but was between those of the control strains BFcf-WT and BFcf-415 (Fig. 10A). The absorption spectrum of the BFcf-WT membrane showed Q_y_ peaks at 865, 803, and 758 nm (Fig. 10B), and pigment analysis revealed the presence of Bchl *a* and Bpheo *a* at a molar ratio of ~2:1 (Fig. 10C). The absorption spectrum of the BFcf-415 membrane (Fig. 10B) was similar to that of BF (Fig. 3D), with Q_y_ peaks at 815 nm and 770 nm (Fig. 6 and 10B). The Q_y_ absorption at 730 nm was well below the peak of 3V-Bchlide *a* (Fig. 10B). Pigment analysis of the BFcf-415 membrane revealed that 3V-Bchl *a* and 3V-Bpheo *a* occurred at a molar ratio of ~2:1 (Fig. 10C).

The BFcf-L67P, BFcf-Y138H, and BFcf-D101N membranes exhibited overlapping spectra of the WT-RC and V-RC (Fig. 10B). The WT-RC was estimated to occur in BFcf-L67P, BFcf-Y138H, and BFcf-D101N at approximately 98%, 79%, and 33% of that of the BFcf-WT value, whereas V-RC occurred at approximately 6%, 16%, and 83% of that of BFcf-415, respectively, assuming that RCs were composed of only one kind of pigment at a stoichiometric ratio of 2:1 (Bchl *a*/Bpheo *a* or 3V-Bchl *a*/3V-Bpheo *a*) (Fig. 10C). The relative ratios of WT-RC to V-RC in BFcf-L67P, BFcf-Y138H, and BFcf-D101N were approximately 19, 5, and 0.5, respectively. Thus, higher levels of BchF activity (Table 1) corresponded to larger amounts of WT-RCs (Fig. 10C), which promotes cell growth (Fig. 10A). However, an RC containing heterogeneous combinations of pigments is possible, and formation of the RC remains to be determined. The levels of both Chlide *a* and 3V-Bchlide *a* from the culture of BFcf-L67P, BFcf-Y138H, and BFcf-D101N were lower than those of BFcf-415 and reflected variation in BchF activity; higher BchF activity corresponded with lower levels of both Chlide *a* and 3V-Bchlide *a* (Fig. 10C).

Collectively, the V-RC formed when 3V-Bchlide *a* accumulated. Although the electron transfer function of the V-RC is inferior to that of the WT-RC, it is photochemically active and supports photoheterotrophic growth of R. sphaeroides. Furthermore, WT- and V-RCs can be coproduced at low levels of BchF activity.

## DISCUSSION

Bchlide *a* must be prenylated by the C_20_ phytol group to form the photosynthetic complex of R. sphaeroides. The BF mutant is deficient in Bchlide *a* biosynthesis but naturally competent in photoheterotrophic growth, which was corroborated by the BCSs harboring the second mutation in *bchF*. The phenotypes of the BCSs were similar to those of BF, resulting in the accumulation of 3V-Bchlide *a*, which was prenylated to form 3V-Bchl *a*.

It is unclear why ROS accumulates during the lag phase in BF growth under photoheterotrophic conditions. 3V-Bchl *a* and 3V-Bpheo *a* are present in cells only when they bind to the V-RC (see Fig. S6I in the supplemental material); therefore, it was assumed that no free phytylated pigment was present in the cell membrane. However, Chlide *a* and 3V-Bchlide *a* immediately accumulated in BF culture under photoheterotrophic growth and reached ~0.03 μM, which was maintained during the lag period. The COR reaction with Chlide *a* generates superoxide at low dissolved oxygen levels (34). However, it is unknown whether the accumulated Chlide *a* and/or 3V-Bchlide *a* is involved in the production of ROS or generation of ROS by other unknown sources.

3HE-Bchlide *a* (C-3-hydroxyethyl, C-7–C-8 single bond) is accumulated by BC, yet it did not react with BchG (Fig. S5D). In contrast, Bchlide *a* (C-3-acetyl, C-7–C-8 single bond) (Fig. 1) and 3V-Bchlide *a* (C-3-vinyl, C-7–C-8 single bond) (Fig. 1) were prenylated by BchG (Fig. S5C and E). An understanding of the prenylation mechanism involving the substrates requires structural elucidation of BchG. Nevertheless, the C-3 functional groups of Bchlide *a* affected the recognition by the enzyme and the catalytic efficiency for prenylation.

The pigments at the redox-active centers P, B, and H of R. sphaeroides RC can be exchanged with various bacteriochlorins with diverse side-functional groups (20, 35). Bacteriochlorins with a central Mg^2+^ are directed into the P- and B-sites, whereas those without Mg^2+^ are directed into the H-site (35). Furthermore, Bchl *a* and Bpheo *a* at the B- and H-sites can be replaced by 3V-Bchl *a* (36) and 3V-Bpheo *a* (37), respectively. The presence of Mg-dechelatase forming Pheo *a* is proposed in plants (38). Mg-dechelatase is not identified in R. sphaeroides; however, specific residues near the H-site of the RC are speculated to affect dechelation and selectively adopt pheophytin species at the site (39). Likewise, 3V-Bpheo *a* may form when 3V-Bchl *a* enters the H-site of the V-RC.

The structural difference between the WT-RC and V-RC lies in the acetyl and vinyl groups at C-3 (Fig. 1) of Bchl *a*/Bpheo *a* and 3V-Bchl *a*/3V-Bpheo *a*, respectively. The oxygen of C-3 acetyl groups (O_C-3_) of B-site Bchl *a* and H-site Bpheo *a* is in contact with phytyl chains of P_A_-site Bchl *a*, connecting the conjugated ring system between the P-, B-, and H-sites, which can facilitate electron transfer in the RC (40). The absence of O_C-3_ in 3V-Bchl *a* results in the loss of such interactions. However, the P_A_→H_A_ electron transfer of the V-RC did not change from that of the WT-RC. Accordingly, O_C-3_ does not affect electron transfer between P and H.

Change in the redox potential of P/P^+^ results in inefficient charge separation and retardation of photosynthetic growth (41). The O_C-3_ of P_A_ forms a hydrogen bond with the 168th His of subunit-L, and the O_C-3_ of P_B_ is in contact with the central Mg^2+^ of P_A_ (42, 43). Replacement of the 168th His of the RC-L subunit with Phe [H(L168)F] destroyed the hydrogen bond between His and the O_C-3_ of P_A_. The *E_m_* of P/P^+^ of the mutant RC with H(L168)F was 95 mV more negative than that of the WT-RC (41). The V-RC lacks the O_C-3_ of P, so the hydrogen bond between the 168th His and P_A_ is absent, as in the mutant RC with H(L168)F. Nonetheless, the *E_m_* of P/P^+^ of the V-RC was 33 mV more positive than that of the WT-RC. This discrepancy may originate from the lack of O_C-3_ in P_B_ of the V-RC, which abolishes its interaction with Mg^2+^ of P_A_. Moreover, the difference in redox potential between Bchl *a* and 3V-Bchl *a* may contribute to the change in the *E_m_* of P/P^+^. The P_680_ Chl *a* of plant photosystem II has a more positive *E_m_* (1.11 to 1.30 V) (44) than the P/P^+^ (0.497 V) (Fig. 9) (33) of the WT-RC, which is ascribed to the difference in the redox potential of Chl *a* and Bchl *a* (41). Likewise, the redox potentials of the P-, B-, and H-sites of the V-RC may be altered to the same extent according to the redox properties of 3V-Bchl *a* and 3V-Bpheo *a*, which diminishes the difference in redox values between H_A_ and Q_A_, resulting in an increased τ for electron transfer P^+^H_A−_Q_A_→P^+^H_A_Q_A−_.

LH1, LH2-1, and LH2-2 of R. sphaeroides do not use 3V-Bchl *a* as cofactors (Fig. 5), indicating that the proteins strictly distinguished acetyl groups from the vinyl group on C-3. The O_C-3_ of the two Bchls *a* in LH1 forms hydrogen bonds with the 43rd Trp of the α subunit and 47th Trp of the β subunit (45, 46). The O_C-3_ of B800 Bchl *a* in LH2-1 forms a hydrogen bond with the 30th Arg of the β subunit, and those of two B850 Bchls *a* form hydrogen bonds with the 44th and 45th Tyr of the α subunit (47, 48). The hydrogen bonds between pigments and protein are critical determinants of spectral properties and pigment selectivity (48). Our findings corroborate the importance of hydrogen bonds involving the O_C-3_ of Bchl *a* for the proper assembly of LH complexes.

The slower growth of BFcf-415 than BFcf-WT may be owing to the lower electron transfer rate of the V-RC than of the WT-RC (Fig. 8 and 10). However, other factors need to be considered. Pigment synthesis is usually harmonized with its integration into the photosynthetic complex. The catalytic efficiency of BchG for the production of 3V-Bchl *a* was 10-fold lower than that of Bchl *a* (Fig. 4E). Moreover, the V-RC was assembled in the presence of accumulated Chlide *a*, which inhibits 3V-Bchl *a* production by BchG (Fig. 4). The binding efficiency of 3V-Bchl *a* for the RC remains unknown. The *in vitro* substitution of the B-site Bchl *a* by 3V-Bchl *a* was incomplete despite the presence of excess exogenous 3V-Bchl *a* (36). Thus, the affinity of 3V-Bchl *a* for the RC is assumed to be lower than that of Bchl *a*, which could affect the formation of the V-RC and, ultimately, cell growth.

Bchl *a* and 3V-Bchl *a* were simultaneously synthesized by BFcf-L67P, BFcf-Y138H, and BFcf-D101N. The six pigment-binding sites of the RC are independently coordinated with bacteriochlorins; therefore, RCs may contain diverse combinations of Bchl *a* (Bpheo *a*) and 3V-Bchl *a* (3V-Bpheo *a*). The possible formations of RCs with heterogeneous pigments remain unclear and may interrupt the formation of the WT-RC and V-RC.

In conclusion, this study described the photoheterotrophic growth of BF through a novel RC. BF synthesizes 3V-Bchl *a*, which is used to form V-RC, not LH complexes. Although the V-RC is inferior to the WT-RC in terms of electron transfer rate, it is still suitable for a light reaction to support photoheterotrophic growth. Furthermore, coproduction of the WT-RC and V-RC was achieved by diminishing cellular BchF activity. The formation of the V-RC without LH complexes may signify that any photosynthetic growth by the V-RC would theoretically be facilitated only at the light intensity saturated for RC activity. Future studies should focus on identifying BchF inhibitors along with their distribution in nature. Further biochemical characterization of the V-RC is required to elucidate the true photosynthetic potential of R. sphaeroides. The formation of V-RCs should be evaluated in related purple nonsulfur photosynthetic bacteria and other photosynthetic bacteria synthesizing Bchl *a*. This could be achieved by assessing substrate specificity for BchG prenylation and characterizing the assembly of pigment-protein complexes.

## MATERIALS AND METHODS

### Bacterial strains and growth conditions.

R. sphaeroides 2.4.1 (49) was cultured in Sis minimal medium (50) at 30°C as previously described (51). Cells were grown aerobically with shaking at 250 rpm; exponentially growing cells were used as an inoculum for anaerobic growth in the dark with 75 mM DMSO as an electron acceptor or photoheterotrophically at a light intensity of 15 W/m^2^ in the presence or absence of 10 mM DMSO as an antioxidant (24). An anaerobic jar (BBL GasPak; BD Biosciences, Franklin Lakes, NJ, USA) was used to select colonies capable of photoheterotrophic growth. Cells were grown semiaerobically with shaking at 50 rpm. E. coli was cultured aerobically at 37°C in Luria-Bertani (LB) medium (52) with shaking at 250 rpm. Antibiotics were added to the medium when necessary at the following concentrations: tetracycline (Tc) and kanamycin (Km) at 1 and 25 μg/mL, respectively, for R. sphaeroides and ampicillin (Ap), Tc, and Km at 50, 20, and 25 μg/mL, respectively, for E. coli.

### Plasmid construction.

All plasmids were constructed in the E. coli strain DH5α *phe* (53). Detailed procedures for plasmid construction are described in Text S1 in the supplemental material.

### Construction of R. sphaeroides mutants.

Detailed procedures for the construction of R. sphaeroides mutants are described in Text S1 in the supplemental material.

### Spectral analysis of culture supernatant and cell membrane.

Detailed procedures for the spectral analysis of culture supernatant and cell membrane are described in Text S1 in the supplemental material.

### Determination of intracellular ROS levels.

General ROS levels were determined as previously described (54). Aliquots of the photoheterotrophic culture were centrifuged at 12,000 × *g* for 1 min at 4°C and resuspended in fresh Sis medium. The oxidation-sensitive fluorescent probe 2,7-dihydrodichlorofluorescein diacetate (H_2_DCFDA; Sigma-Aldrich, St. Louis, MO, USA) was added to the cell suspension at the final concentration of 10 μM. Samples were incubated at 30°C for 30 min in the dark, followed by fluorescence measurement (excitation at 492 nm, emission at 525 nm) using a microplate reader (EnSpire; PerkinElmer, Waltham, MA, USA). Samples were measured in triplicate, and those without H_2_DCFDA were used as background controls.

### In-gel activity staining of SOD and catalase.

Detailed procedures for the in-gel activity staining of SOD and catalase are described in Text S1 in the supplemental material.

### Extraction and analysis of the pigments from membranes or cell lysates.

The cell membrane (0.1 mL) or cell lysate (0.1 mL) prepared as described in Text S1 was mixed with 0.9 mL extraction solution (acetone/methanol ratio = 7:2, vol/vol), followed by centrifugation at 12,000 × *g* for 5 min at 4°C to remove insoluble materials. *n*-Hexane (0.5 mL) was added for pigment extraction, and the samples were mixed by vigorous shaking. Two phases were obtained after centrifugation at 4,000 × *g* for 5 min at 4°C. The upper phase containing bacteriochlorin with the C_20_ group (25) was dried under an N_2_ stream and dissolved with 70% acetone for HPLC analysis. Pigments were determined by HPLC as previously described (55) with some modifications. HPLC was performed using a Shimadzu LC-6AD dual pump system equipped with a Zorbax octyldecyl silane (ODS) column (Agilent Technologies, Santa Clara, CA, USA; particle size of 5 μm, diameter by length of 4.6 by 250 mm) and a UV-visible (UV-Vis) absorbance detector. Samples were eluted by linear gradients consisting of an acetone (A)-water (B) mixture for 29 min at 1 mL/min, and absorbance was monitored at the appropriate wavelength for each pigment. The gradient was as follows: 0 min, 70% A; 2 min, 70% A; 4 min, 82% A; 15 min, 88% A; 19 min, 100% A; 24 min, 100% A; 29 min, 70% A. Mass spectrometry was performed on the major peaks with retention times between 15 and 27 min (bacteriochlorin pigments with the C_20_ group) that were pooled by a fraction collector (FRC-10A; Shimadzu). *n*-Hexane was added to the pooled fraction, and the upper layer was collected. The pigment sample was dried under an N_2_ stream and finally dissolved in methanol. Samples were analyzed using cold-spray ionization and time of flight mass spectrometry (TOF-MS; JMS-T100LP 4G; JEOL, Tokyo, Japan) to determine the mass of molecular ions.

### Preparation of AtChlase.

C-terminally Strep-tagged Arabidopsis thaliana chlorophyllase (AtChlase) was overexpressed from actively growing recombinant E. coli BL-Chlase (optical density at 600 nm [OD_600_] = 0.6) (Table S1) with anhydrotetracycline (0.2 μg/mL). Cells were grown for a further 12 h and harvested by centrifugation at 6,000 × *g* for 5 min at 4°C. The cell pellet was resuspended with Buffer-SW (100 mM Tris-Cl, pH 8.0, 150 mM NaCl). Cells were disrupted by sonication (Branson Sonifier model 250; Danbury, CT, USA) on ice for 5 min, three times. The lysate was centrifuged at 8,000 × *g* for 15 min at 4°C, and the supernatant was loaded onto a Strep-Tactin resin column (IBA Life Sciences, Göttingen, Germany). The resin was washed with Buffer-SW, and AtChlase was eluted with Buffer-SW supplemented with 2.5 mM desthiobiotin. The eluate was used as the AtChlase.

### Pigment preparation.

Bchl *a* and Chl *a* were purchased from Sigma-Aldrich. Bchl *a*_GG_ was purified from R. sphaeroides mutant BP (Table S1), which was photoheterotrophically cultured for 5 days. 3V-Bchl *a* was purified from BF (Table S1) that was photoheterotrophically grown in the presence of 10 mM DMSO for 5 days. 3V-Bchl *a*_GG_ was purified from BFP (Table S1) that was semiaerobically grown for 5 days. Pigments were isolated from the corresponding cells using dioxane and Sepharose CL-6B as previously described (56). Bchlide *a*, Chlide *a*, and 3V-Bchlide *a* were prepared from Bchl *a*, Chl *a*, and 3V-Bchl *a*, respectively, following enzymatic digestion of the phytyl moiety using AtChlase (55). Pigment concentrations were determined using the following extinction coefficients (per millimolar per centimeter): Bchl *a*, 83.9 at 771 nm; Chl *a*, 85.6 at 662 nm; 3V-Bchl *a*, 82.5 at 734 nm; Bchlide *a*, 42.1 at 773 nm; Chlide *a*, 54.1 at 665 nm; and 3V-Bchlide *a*, 44.7 at 734 nm (25, 57). The extinction coefficient of Bchl *a*_GG_ was comparable to that of Bchl *a* because equivalent levels of Bchlide *a* were produced when either Bchl *a*_GG_ or Bchl *a* at the same absorbance was digested with AtChlase. The same was true for 3V-Bchl *a*_GG_ and 3V-Bchl *a*.

### Kinetic analysis of BchG.

R. sphaeroides BchG was overexpressed in E. coli BL-Rsb (Table S1) cultured aerobically in 30 mL LB medium supplemented with Km at 37°C until reaching an OD_600_ of 0.6. Isopropyl-β-d-thiogalactopyranoside (IPTG) was added at 1 mM to induce protein overexpression, followed by cultivation under the same conditions for another 12 h. Cells were harvested by centrifugation at 6,000 × *g* for 5 min at 4°C and washed once with phosphate-buffered saline (PBS). The cell pellet was resuspended in 5 mL Buffer-G (120 mM potassium acetate, 10 mM magnesium acetate, 50 mM HEPES-KOH, pH 7.6, 14 mM beta-mercaptoethanol, and 10% glycerol) (55) and lysed by sonication. Cell lysates were obtained by centrifugation at 8,000 × *g* for 5 min at 4°C and used as an enzyme source.

BchG activity was assayed as previously described (55). Cell lysate (1 mg total protein) was added to 0.2 mL Buffer-G supplemented with 0.5 mM ATP, 50 μM GGPP (Sigma-Aldrich) or PPP (Indofine Chemical Company, Hillsborough, NJ, USA), and 10 to 80 μM Bchlide *a* or 3V-Bchlide *a*. The inhibition of BchG activity by Chlide *a* was analyzed at its various levels between 10 μM and 40 μM. The reaction was performed at 30°C for 3 h and stopped by adding 0.8 mL acetone. The reaction mixture was centrifuged at 12,000 × *g* for 5 min at 4°C, and the supernatant was filtered through a 0.45-μm filter. The reaction products were quantified using the HPLC pigment analysis by monitoring the absorbance at 771 nm for Bchl *a* (or Bchl *a*_GG_) and 734 nm for 3V-Bchl *a* (or 3V-Bchl *a*_GG_). Standard curves for Bchl *a*, Bchl *a*_GG_, 3V-Bchl *a*, and 3V-Bchl *a*_GG_ were constructed using known amounts of pigment standards. The data sets of *V*_0_ were fitted to a competitive inhibition model by nonlinear regression using SigmaPlot ver. 14 (Systat Software, San Jose, CA, USA). Data were obtained from three independent experiments. BchG with a C-terminal His_6_ tag (BchG-His_6_, 34.0 kDa) was quantified as previously described to determine *k*_cat_ (58). PpfC-His_6_ of V. vulnificus (38.0 kDa) (28) was used as the protein standard. E. coli lysates containing BchG were loaded onto a 12% SDS-polyacrylamide gel with known amounts of PpfC-His_6_, which were subjected to Western immunoblot analysis using an anti-His_6_ tag antibody (MBL, Woburn, MA, USA). Immunoblotted protein bands with correct sizes were quantified by densitometric scanning using Image J (59).

### Purification of His_6_-tagged photosynthetic complexes from R. sphaeroides.

Wcfh-puhA, Wcfh-puf, Wcfh-puc1, Wcfh-puc2, Wcfh-415, BFcfh-puhA, BFcfh-puf, BFcfh-puc1, BFcfh-puc2, and BFcfh-415 (Table S1) were cultured semiaerobically for 5 days in Sis medium supplemented with 0.25% yeast extract to promote cell growth. His_6_-tagged photosynthetic complexes were purified as previously described for RC purification (60, 61). The eluates from His tag affinity chromatography were dialyzed against Buffer-R containing 10 mM Tris-Cl, pH 8.0, and 0.05% LDAO for 24 h at 4°C and analyzed for spectral profiles.

### Pigment analysis of the purified RC.

Wcfh-puhA and BFcfh-puhA (Table S1) were grown semiaerobically in Sis medium supplemented with 0.25% yeast extract or photoheterotrophically in Sis medium with 10 mM DMSO for 5 days. The WT-RC and V-RC were purified from Wcfh-puhA and BFcfh-puhA, respectively, as previously described (60, 61). Aliquots (0.1 mL) of the WT-RC (10 μg) and V-RC (16 μg) were digested with trypsin (0.5 mg/mL; 5,000 BAEE U/mL) and α-chymotrypsin (0.5 mg/mL; 20 μmol BTEE/min/mL) for 1 h at 37°C, and the membrane was disintegrated with 0.9 mL extraction solution, followed by centrifugation at 12,000 × *g* for 5 min at 4°C to remove insoluble materials. Pigments were extracted using *n*-hexane and dried under an N_2_ stream. The samples were dissolved in 80% acetone. Bacteriochlorins from the RC were quantified by HPLC pigment analysis. Bpheo *a* and 3V-Bpheo *a* were quantified by measuring their absorbance at 749 nm and 719 nm, respectively. Standard molecules of Bpheo *a* and 3V-Bpheo *a* were obtained by the dechelation of Mg^2+^ from Bchl *a* and 3V-Bchl *a*, respectively, involving treatment with 0.2 M HCl as previously described (62). Carotenoids were quantified using HPLC as previously described (63). Samples were injected onto a Zorbax ODS column with an isocratic flow of an acetonitrile–methanol–2-propanol mixture (50:30:20, vol/vol/vol) at 1 mL/min for 50 min, and the absorbance was monitored at 482 nm. The major carotenoid peaks were pooled by a fraction collector, dried under an N_2_ stream, and dissolved in extraction solution. Carotenoid concentrations were determined by UV-Vis spectrophotometry using extinction coefficients (per millimolar per centimeter) of 123.6 for SO at 482 nm and 153.4 for SE at 456 nm (64). The amount of RC polypeptide was quantified by Western immunoblotting of PuhA-His_6_ using an anti-His_6_ tag antibody in the same manner as that for BchG-His_6_ quantification. PpfC-His_6_ was used as the protein standard.

### Broadband transient absorption spectroscopy.

Transient absorption spectra were measured using femtosecond laser pulses based on a pump-probe scheme as described in previous studies (65, 66). The output pulses at the wavelength of 800 nm from a Ti-sapphire amplified laser (Legend Elite; Coherent, Santa Clara, CA, USA) were divided into the pump and probe parts. On the pump arm, the 800-nm laser pulses with the bandwidth of 21 nm in full width at half maximum (FWHM) were used as a pump pulse and were not compressed further. At the sample position, the pulse energies of the pump pulses were adjusted to 510 nJ (~0.46 mJ/mm^2^). On the probe arm, the 800-nm laser pulses were sent into a 4-mm *c*-cut ytterbium aluminum garnet (YAG) window and converted into a white light continuum spanning from visible to near-infrared (NIR) wavelengths by self-phase modulation. The visible-to-NIR portion (500 to 1,300 nm generated by the YAG window) of the white light continuum was used as the supercontinuum probe without the chirp compression, and the fundamental pulses were eliminated by a 725-nm short-pass filter. The pump pulses were delayed in time with respect to the probe pulses using a motorized translation stage (M-ILS150HA; Newport, Irvine, CA, USA). The transient absorption spectra were collected by a spectrometer (SR303i; Andor, Belfast, UK) equipped with a Si charge-coupled device (CCD) (Newton DU970P; Andor) for the visible probe and an InGaAs CCD (DU491A-1.7; Andor) for the NIR probe. In all measurements, the linear polarization of the pump pulses was set to be a magic angle (54.7°) relative to that of the probe pulses using a half waveplate and an analyzer. The instrument response function of the transient absorption measurement was estimated to be about 100 fs. The final solutions of WT-RC and V-RC, which were prepared from the semiaerobically grown cells, were placed in the 2-mm-thick quartz cells, and the optical densities of the solution were adjusted to be ~0.90 for WT-RC and ~0.58 for V-RC at 800 nm in Buffer-R. To avoid the long-term sample damage induced by laser pulses, the sample solutions were mixed up occasionally by a magnetic stirrer after the short-term measurements. The transient absorption spectra of WT-RC and V-RC were collected in the time range up to 650 ps.

### Electrochemical titration of P/P^+^ in RC.

The electrochemical titrations of the RCs were performed as previously described with some modifications (41). Buffer-T (20 mM Tris-Cl, pH 8.0, 60 mM KCl, 0.05% LDAO) was degassed and equilibrated by argon gas. After the *A*_865_ of WT-RC and *A*_815_ of V-RC were diluted to 0.5 to 0.6 in Buffer-T, potassium ferrocyanide was added at the final concentration of 0.72 mM to each analyte solution. Each solution was oxidatively titrated upon adding the aliquots of potassium permanganate, by which the portion of ferrocyanide was oxidized to ferricyanide, resulting in the change in the redox potential of the solution. Meanwhile, the *A*_865_ and *A*_815_ were recorded to monitor the reduction state of P/P^+^ in the WT-RC and V-RC, respectively. Simultaneously, the solution potential was recorded at each titration point by a potentiometer equipped with an Ag|AgCl|KCl (saturated) electrode as a reference. The data were fitted to the Nernst equation:

$$\frac{\left[\text{P}\right]}{\left[{\text{P}}^{+}\right]}=\frac{A-A_{\text{Oxd}}}{A_{\text{Red}}-A}\;=\exp\left[0.03894\;\left(E_{m}-E\right)\right]$$

where *A* is the recorded absorbance at 865 nm or 815 nm, *A*_Oxd_ is the absorbance at the fully oxidized point, *A*_Red_ is the absorbance at the fully reduced point, *E_m_* is the midpoint potential, and *E* is the recorded solution potential. Parameter *n* was set to 1 from the number of electrons transferred in the ferri-ferrocyanide redox pair.

### Kinetic analysis of BchF.

WT and mutant BchFs were overexpressed in the E. coli strains BL-WT, BL-L67P, BL-Y138H, and BL-D101N (Table S1). Cells were grown aerobically at 37°C in 30 mL LB medium supplemented with Km until the OD_600_ reached 0.6. Then, IPTG was added to a final concentration of 1 mM for protein overexpression, followed by cultivation for another 6 h. Cells were harvested by centrifugation at 6,000 × *g* for 5 min at 4°C and washed once with Buffer-F (25 mM Tris-Cl, pH 7.8, 7.5 mM NaCl). The cell pellets were resuspended in 5 mL Buffer-F and lysed by sonication. Cell lysates for enzyme assay were collected after centrifugation at 8,000 × *g* for 10 min at 4°C.

The enzyme activity of BchF was determined as previously described (67). Cell lysate (0.2 mg total protein) containing C-terminally His_6_-tagged BchF (BchF-His_6_) was added to 0.2 mL Buffer-F containing 10% DMSO. The reaction was initiated by adding various concentrations of Chlide *a* (50 to 400 μM) and 3V-Bchlide *a* (50 to 400 μM). The reaction mixture was incubated at 30°C for 1 h (BchF^WT^) or 3 h (BchF^L67P^, BchF^Y138H^, and BchF^D101N^). The reaction was stopped by adding 0.8 mL acetone and by centrifugation at 12,000 × *g* for 5 min at 4°C. The supernatant was filtered through a 0.45-μm filter and injected into the HPLC system equipped with an UltraSep ES 100 RP 8E column (Dr. Maisch GmbH, Ammerbuch, Germany; particle size of 5 μm, diameter by length of 4.6 by 250 mm) (68). The samples were eluted using a linear gradient of acetonitrile, and water containing 0.005% triethylamine. The acetonitrile concentration was linearly increased from 25% to 100% over 27 min at 1 mL/min. The products (3HE-Chlide *a* and 3HE-Bchlide *a*) were pooled and quantified using a UV-Vis spectrophotometer with an experimentally determined extinction coefficient: Chlide *a* was hydrated with cell lysate containing BchF^WT^-His_6_ (lysate of 1 mg total protein) at 30°C for 6 h, and the product and remaining substrate were separated by HPLC and quantified for Chlide *a*, from which the extinction coefficient of 3HE-Chlide *a* was estimated as 41.6 mM^−1^ cm^−1^ at 662 nm (in 80% acetone). Similarly, the extinction coefficient of 3HE-Bchlide *a* was determined using the reverse reaction of BchC: Bchlide *a* was reduced by BchC-His_6_ (10 μM and purified from BL-BchC [Table S1]) in the presence of 1 mM NADH in 50 mM Tris-Cl (pH 8.0) at 30°C for 30 min (68), from which the extinction coefficient of 3HE-Bchlide *a* was determined to be 34.2 mM^−1^ cm^−1^ at 715 nm (in 80% acetone).

The data sets of initial rates (*V*_0_) were fitted to the Michaelis-Menten equation by nonlinear regression using SigmaPlot ver. 14 (Systat Software). Data were obtained from three independent experiments. BchF (WT and mutant enzymes)-His_6_ (20.1 kDa) in E. coli lysates was quantified in the same manner as that used for the determination of BchG-His_6_ to determine *k*_cat_, and C-terminally His_6_-tagged carbonic anhydrase from R. sphaeroides (RsCA-His_6_, 25.0 kDa) was purified from E. coli BL-RsCA (Table S1) and used as the protein standard.

## References

[1] Proctor MS, Sutherland GA, Canniffe DP, Hitchcock A. 2022. The terminal enzymes of (bacterio)chlorophyll biosynthesis. R Soc Open Sci 9:211903. doi:10.1098/rsos.211903. 35573041 PMC9066304

[2] Pudek MR, Richards WR. 1975. A possible alternate pathway of bacteriochlorophyll biosynthesis in a mutant of Rhodopseudomonas sphaeroides. Biochemistry 14:3132–3137. doi:10.1021/bi00685a015. 1080053

[3] Bollivar DW, Suzuki JY, Beatty JT, Dobrowolski JM, Bauer CE. 1994. Directed mutational analysis of bacteriochlorophyll a biosynthesis in Rhodobacter capsulatus. J Mol Biol 237:622–640. doi:10.1006/jmbi.1994.1260. 8158642

[4] Struck A, Beese D, Cmiel E, Fischer M, Müller A, Schäfer W, Scheer H. 1990. Modified bacterial reaction centers: 3. Chemically modified chromophores at sites BA, BB and HA, HB, p 313–326. *In* Michel-Beyerle M-E (ed), Reaction centers of photosynthetic bacteria. Springer, Berlin, Germany.

[5] Ortega-Ramos M, Canniffe DP, Radle MI, Hunter CN, Bryant DA, Golbeck JH. 2018. Engineered biosynthesis of bacteriochlorophyll g_F_ in Rhodobacter sphaeroides. Biochim Biophys Acta Bioenerg 1859:501–509. doi:10.1016/j.bbabio.2018.02.006. 29496394

[6] Davis J, Donohue TJ, Kaplan S. 1988. Construction, characterization, and complementation of a Puf^−^ mutant of *Rhodobacter sphaeroides*. J Bacteriol 170:320–329. doi:10.1128/jb.170.1.320-329.1988. 3257209 PMC210645

[7] Kiley PJ, Kaplan S. 1987. Cloning, DNA sequence, and expression of the *Rhodobacter sphaeroides* light-harvesting B800-850-α and B800-850-β genes. J Bacteriol 169:3268–3275. doi:10.1128/jb.169.7.3268-3275.1987. 3036782 PMC212379

[8] Zeng X, Choudhary M, Kaplan S. 2003. A second and unusual pucBA operon of *Rhodobacter sphaeroides* 2.4.1: genetics and function of the encoded polypeptides. J Bacteriol 185:6171–6184. doi:10.1128/JB.185.20.6171-6184.2003. 14526029 PMC225038

[9] Jolchine G, Reiss-Husson F. 1975. Studies on pigments and lipids in Rhodopseudomonas spheroides Y reaction center. FEBS Lett 52:33–36. doi:10.1016/0014-5793(75)80631-4. 1079006

[10] Jolchine G, Reiss-Husson F. 1974. Comparative studies on two reaction center preparations from Rhodopseudomonas spheroides Y. FEBS Lett 40:5–8. doi:10.1016/0014-5793(74)80881-1. 4546760

[11] Cogdell RJ, Parson WW, Kerr MA. 1976. The type, amount, location, and energy transfer properties of the carotenoid in reaction centers from Rhodopseudomonas sphaeroides. Biochim Biophys Acta Bioenerg 430:83–93. doi:10.1016/0005-2728(76)90224-3. 1083252

[12] Laible PD, Hanson DK, Buhrmaster JC, Tira GA, Faries KM, Holten D, Kirmaier C. 2020. Switching sides—reengineered primary charge separation in the bacterial photosynthetic reaction center. Proc Natl Acad Sci U S A 117:865–871. doi:10.1073/pnas.1916119117. 31892543 PMC6969525

[13] Ishikita H, Galstyan A, Knapp E-W. 2007. Redox potential of the non-heme iron complex in bacterial photosynthetic reaction center. Biochim Biophys Acta Bioenerg 1767:1300–1309. doi:10.1016/j.bbabio.2007.08.004. 17936717

[14] Franken EM, Shkuropatov AY, Francke C, Neerken S, Gast P, Shuvalov VA, Hoff AJ, Aartsma TJ. 1997. Reaction centers of Rhodobacter sphaeroides R-26 with selective replacement of bacteriopheophytin by pheophytin a: I. Characterisation of steady-state absorbance and circular dichroism, and of the P^+^QA- state. Biochim Biophys Acta Bioenerg 1319:242–250. doi:10.1016/S0005-2728(96)00165-X.

[15] Franken EM, Shkuropatov AY, Francke C, Neerken S, Gast P, Shuvalov VA, Hoff AJ, Aartsma TJ. 1997. Reaction centers of Rhodobacter sphaeroides R-26 with selective replacement of bacteriopheophytin a by pheophytin a: II. Temperature dependence of the quantum yield of P^+^QA- and ^3^P formation. Biochim Biophys Acta Bioenerg 1321:1–9. doi:10.1016/S0005-2728(97)00039-X.

[16] Shkuropatov AY, Shuvalov VA. 1993. Electron transfer in pheophytin a-modified reaction centers from Rhodobacter sphaeroides (R-26). FEBS Lett 322:168–172. doi:10.1016/0014-5793(93)81561-D. 8482386

[17] Shkuropatov AY, Proskuryakov II, Shkuropatova VA, Zvereva MG, Shuvalov VA. 1994. Formation of charge separated state P^+^Q_A_- and triplet state ^3^P at low temperature in Rhodobacter sphaeroides (R-26) reaction centers in which bacteriopheophytin a is replaced by plant pheophytin a. FEBS Lett 351:249–252. doi:10.1016/0014-5793(94)00843-4. 8082774

[18] Kennis JTM, Shkuropatov AY, Stokkum IHM, Gast P, Hoff AJ, Shuvalov VA, Aartsma TJ. 1997. Formation of a long-lived P^+^B_A_^−^ state in plant pheophytin-exchanged reaction centers of Rhodobacter sphaeroides R26 at low temperature. Biochemistry 36:16231–16238. doi:10.1021/bi9712605. 9405057

[19] Jaschke PR, Hardjasa A, Digby EL, Hunter CN, Beatty JT. 2011. A bchD (magnesium chelatase) mutant of Rhodobacter sphaeroides synthesizes zinc bacteriochlorophyll through novel zinc-containing intermediates. J Biol Chem 286:20313–20322. doi:10.1074/jbc.M110.212605. 21502322 PMC3121458

[20] Lin S, Jaschke PR, Wang H, Paddock M, Tufts A, Allen JP, Rosell FI, Mauk AG, Woodbury NW, Beatty JT. 2009. Electron transfer in the Rhodobacter sphaeroides reaction center assembled with zinc bacteriochlorophyll. Proc Natl Acad Sci U S A 106:8537–8542. doi:10.1073/pnas.0812719106. 19439660 PMC2689001

[21] Neupane B, Jaschke P, Saer R, Beatty JT, Reppert M, Jankowiak R. 2012. Electron transfer in Rhodobacter sphaeroides reaction centers containing Zn-bacteriochlorophylls: a hole-burning study. J Phys Chem B 116:3457–3466. doi:10.1021/jp300304r. 22324747

[22] Saer RG, Pan J, Hardjasa A, Lin S, Rosell F, Mauk AG, Woodbury NW, Murphy MEP, Beatty JT. 2014. Structural and kinetic properties of Rhodobacter sphaeroides photosynthetic reaction centers containing exclusively Zn-coordinated bacteriochlorophyll as bacteriochlorin cofactors. Biochim Biophys Acta Bioenerg 1837:366–374. doi:10.1016/j.bbabio.2013.11.015. 24316146

[23] Coomber SA, Chaudhri M, Connor A, Britton G, Hunter CN. 1990. Localized transposon Tn5 mutagenesis of the photosynthetic gene cluster of Rhodobacter sphaeroides. Mol Microbiol 4:977–989. doi:10.1111/j.1365-2958.1990.tb00670.x. 2170816

[24] Wasil M, Halliwell B, Grootveld M, Moorhouse CP, Hutchison DCS, Baum H. 1987. The specificity of thiourea, dimethylthiourea and dimethyl sulphoxide as scavengers of hydroxyl radicals. Their protection of α1-antiproteinase against inactivation by hypochlorous acids. Biochem J 243:867–870. doi:10.1042/bj2430867. 2821995 PMC1147938

[25] Tanaka K, Kakuno T, Yamashita J, Horio T. 1982. Purification and properties of chlorophyllase from greened rye seedlings. J Biochem 92:1763–1773. doi:10.1093/oxfordjournals.jbchem.a134106. 6819291

[26] Teramura M, Harada J, Mizoguchi T, Yamamoto K, Tamiaki H. 2016. In vitro assays of BciC showing C13^2^-demethoxycarbonylase activity requisite for biosynthesis of chlorosomal chlorophyll pigments. Plant Cell Physiol 57:1048–1057. doi:10.1093/pcp/pcw045. 26936794

[27] Kim E-J, Lee JK. 2010. Competitive inhibitions of the chlorophyll synthase of *Synechocystis* sp. strain PCC 6803 by bacteriochlorophyllide *a* and the bacteriochlorophyll synthase of *Rhodobacter sphaeroides* by chlorophyllide *a*. J Bacteriol 192:198–207. doi:10.1128/JB.01271-09. 19880605 PMC2798255

[28] Kim H, Kim H, Lee JK. 2018. Biochemical characterization of protoporphyrinogen dehydrogenase and protoporphyrin ferrochelatase of Vibrio vulnificus and the critical complex formation between these enzymes. Biochim Biophys Acta Gen Subj 1862:2674–2687. doi:10.1016/j.bbagen.2018.08.012. 30251658

[29] Cogdell RJ. 1983. Photosynthetic reaction centers. Annu Rev Plant Physiol 34:21–45. doi:10.1146/annurev.pp.34.060183.000321.

[30] Yeliseev AA, Eraso JM, Kaplan S. 1996. Differential carotenoid composition of the B875 and B800-850 photosynthetic antenna complexes in *Rhodobacter sphaeroides* 2.4.1: involvement of spheroidene and spheroidenone in adaptation to changes in light intensity and oxygen availability. J Bacteriol 178:5877–5883. doi:10.1128/jb.178.20.5877-5883.1996. 8830681 PMC178441

[31] Kirmaier C, Holten D. 1987. Primary photochemistry of reaction centers from the photosynthetic purple bacteria. Photosynth Res 13:225–260. doi:10.1007/BF00029401. 24435821

[32] Holten D, Hoganson C, Windsor MW, Schenck CC, Parson WW, Migus A, Fork RL, Shank CV. 1980. Subpicosecond and picosecond studies of electron transfer intermediates in Rhodopseudomonas sphaeroides reaction centers. Biochim Biophys Acta Bioenerg 592:461–477. doi:10.1016/0005-2728(80)90092-4. 6968221

[33] Williams JC, Alden RG, Murchison HA, Peloquin JM, Woodbury NW, Allen JP. 1992. Effects of mutations near the bacteriochlorophylls in reaction centers from Rhodobacter sphaeroides. Biochemistry 31:11029–11037. doi:10.1021/bi00160a012. 1445841

[34] Kim E-J, Kim J-S, Lee I-H, Rhee HJ, Lee JK. 2008. Superoxide generation by chlorophyllide a reductase of Rhodobacter sphaeroides. J Biol Chem 283:3718–3730. doi:10.1074/jbc.M707774200. 18079120

[35] Deisenhofer J, Norris JR. 1993. The photosynthetic reaction center, vol II, 1st ed. Academic Press, Inc, San Diego, CA.

[36] Struck A, Cmiel E, Katheder I, Scheer H. 1990. Modified reaction centers from Rhodobacter sphaeroides R26. 2: Bacteriochlorophylls with modified C-3 substituents at sites B_A_ and B_B_. FEBS Lett 268:180–184. doi:10.1016/0014-5793(90)81003-7. 2384154

[37] Meyer M, Scheer H. 1995. Reaction centers of Rhodobacter sphaeroides R26 containing C-3 acetyl and vinyl (bacterio)pheophytins at sites H_A,B_. Photosynth Res 44:55–65. doi:10.1007/BF00018296. 24307025

[38] Matile P, Hörtensteiner S, Thomas H, Kraütler B. 1996. Chlorophyll breakdown in senescent leaves. Plant Physiol 112:1403–1409. doi:10.1104/pp.112.4.1403. 12226455 PMC158071

[39] Jaschke PR, Beatty JT. 2007. The photosystem of Rhodobacter sphaeroides assembles with zinc bacteriochlorophyll in a bchD (magnesium chelatase) mutant. Biochemistry 46:12491–12500. doi:10.1021/bi701407k. 17910480

[40] Yeates TO, Komiya H, Chirino A, Rees DC, Allen JP, Feher G. 1988. Structure of the reaction center from Rhodobacter sphaeroides R-26 and 2.4.1: protein-cofactor (bacteriochlorophyll, bacteriopheophytin, and carotenoid) interactions. Proc Natl Acad Sci U S A 85:7993–7997. doi:10.1073/pnas.85.21.7993. 3186702 PMC282340

[41] Lin X, Murchison HA, Nagarajan V, Parson WW, Allen JP, Williams JC. 1994. Specific alteration of the oxidation potential of the electron donor in reaction centers from Rhodobacter sphaeroides. Proc Natl Acad Sci U S A 91:10265–10269. doi:10.1073/pnas.91.22.10265. 7937938 PMC45000

[42] Allen JP, Williams JC. 1995. Relationship between the oxidation potential of the bacteriochlorophyll dimer and electron transfer in photosynthetic reaction centers. J Bioenerg Biomembr 27:275–283. doi:10.1007/BF02110097. 8847341

[43] Ermler U, Fritzsch G, Buchanan SK, Michel H. 1994. Structure of the photosynthetic reaction centre from Rhodobacter sphaeroides at 2.65 Å resolution: cofactors and protein-cofactor interactions. Structure 2:925–936. doi:10.1016/S0969-2126(94)00094-8. 7866744

[44] Ishikita H, Loll B, Biesiadka J, Saenger W, Knapp E-W. 2005. Redox potentials of chlorophylls in the photosystem II reaction center. Biochemistry 44:4118–4124. doi:10.1021/bi047922p. 15751989

[45] Sturgis JN, Olsen JD, Robert B, Hunter CN. 1997. Functions of conserved tryptophan residues of the core light-harvesting complex of Rhodobacter sphaeroides. Biochemistry 36:2772–2778. doi:10.1021/bi962524a. 9062104

[46] Qian P, Papiz MZ, Jackson PJ, Brindley AA, Ng IW, Olsen JD, Dickman MJ, Bullough PA, Hunter CN. 2013. Three-dimensional structure of the Rhodobacter sphaeroides RC-LH1-PufX complex: dimerization and quinone channels promoted by PufX. Biochemistry 52:7575–7585. doi:10.1021/bi4011946. 24131108

[47] Qian P, Swainsbury DJK, Croll TI, Castro-Hartmann P, Divitini G, Sader K, Hunter CN. 2021. Cryo-EM structure of the Rhodobacter sphaeroides light-harvesting 2 complex at 2.1 Å. Biochemistry 60:3302–3314. doi:10.1021/acs.biochem.1c00576. 34699186 PMC8775250

[48] Swainsbury DJK, Faries KM, Niedzwiedzki DM, Martin EC, Flinders AJ, Canniffe DP, Shen G, Bryant DA, Kirmaier C, Holten D, Hunter CN. 2019. Engineering of B800 bacteriochlorophyll binding site specificity in the Rhodobacter sphaeroides LH2 antenna. Biochim Biophys Acta Bioenerg 1860:209–223. doi:10.1016/j.bbabio.2018.11.008. 30414933 PMC6358721

[49] Cohen-Bazire G, Sistrom WR, Stanier RY. 1957. Kinetic studies of pigment synthesis by non-sulfur purple bacteria. J Cell Comp Physiol 49:25–68. doi:10.1002/jcp.1030490104. 13416343

[50] Sistrom WR. 1960. A requirement for sodium in the growth of Rhodopseudomonas spheroides. J Gen Microbiol 22:778–785. doi:10.1099/00221287-22-3-778. 14447230

[51] Donohue TJ, McEwan AG, Kaplan S. 1986. Cloning, DNA sequence, and expression of the *Rhodobacter sphaeroides* cytochrome *c*_2_ gene. J Bacteriol 168:962–972. doi:10.1128/jb.168.2.962-972.1986. 3023293 PMC213578

[52] Green MR, Sambrook J. 2012. Molecular cloning: a laboratory manual, 4th ed. Cold Spring Harbor Laboratory Press, Cold Spring Harbor, NY.

[53] Eraso JM, Kaplan S. 1994. *prrA*, a putative response regulator involved in oxygen regulation of photosynthesis gene expression in *Rhodobacter sphaeroides*. J Bacteriol 176:32–43. doi:10.1128/jb.176.1.32-43.1994. 8282708 PMC205011

[54] Licht MK, Nuss AM, Volk M, Konzer A, Beckstette M, Berghoff BA, Klug G. 2020. Adaptation to photooxidative stress: common and special strategies of the Alphaproteobacteria Rhodobacter sphaeroides and Rhodobacter capsulatus. Microorganisms 8:283. doi:10.3390/microorganisms8020283. 32093084 PMC7074977

[55] Oster U, Bauer CE, Rudiger W. 1997. Characterization of chlorophyll a and bacteriochlorophyll a synthases by heterologous expression in Escherichia coli. J Biol Chem 272:9671–9676. doi:10.1074/jbc.272.15.9671. 9092496

[56] Omata T, Murata N. 1983. Preparation of chlorophyll a, chlorophyll b and bacteriochlorophyll a by column chromatography with DEAE-Sepharose CL-6B and Sepharose CL-6B. Plant Cell Physiol 24:1093–1100.

[57] Nomata J, Mizoguchi T, Tamiaki H, Fujita Y. 2006. A second nitrogenase-like enzyme for bacteriochlorophyll biosynthesis: reconstitution of chlorophyllide a reductase with purified X-protein (BchX) and YZ-protein (BchY-BchZ) from Rhodobacter capsulatus. J Biol Chem 281:15021–15028. doi:10.1074/jbc.M601750200. 16571720

[58] Yan X, Fan Y, Wei W, Wang P, Liu Q, Wei Y, Zhang L, Zhao G, Yue J, Zhou Z. 2014. Production of bioactive ginsenoside compound K in metabolically engineered yeast. Cell Res 24:770–773. doi:10.1038/cr.2014.28. 24603359 PMC4042165

[59] Schneider CA, Rasband WS, Eliceiri KW. 2012. NIH Image to ImageJ: 25 years of image analysis. Nat Methods 9:671–675. doi:10.1038/nmeth.2089. 22930834 PMC5554542

[60] Pan J, Saer RG, Lin S, Guo Z, Beatty JT, Woodbury NW. 2013. The protein environment of the bacteriopheophytin anion modulates charge separation and charge recombination in bacterial reaction centers. J Phys Chem B 117:7179–7189. doi:10.1021/jp400132k. 23688348

[61] Abresch EC, Axelrod HLA, Beatty JT, Johnson JA, Nechushtai R, Paddock ML. 2005. Characterization of a highly purified, fully active, crystallizable RC–LH1–PufX core complex from Rhodobacter sphaeroides. Photosynth Res 86:61–70. doi:10.1007/s11120-005-5106-z. 16172926

[62] Mazaki H, Watanabe T. 1988. Pheophytinization of chlorophyll a and chlorophyll a′ in aqueous acetone. Bull Chem Soc Jpn 61:2969–2970. doi:10.1246/bcsj.61.2969.

[63] Qiang S, Su AP, Li Y, Chen Z, Hu CY, Meng YH. 2019. Elevated β-carotene synthesis by the engineered Rhodobacter sphaeroides with enhanced CrtY expression. J Agric Food Chem 67:9560–9568. doi:10.1021/acs.jafc.9b02597. 31368704

[64] Shneour EA. 1962. Carotenoid pigment conversion in Rhodopseudomonas spheroides. Biochim Biophys Acta 62:534–540. doi:10.1016/0006-3002(62)90235-4. 13912201

[65] Kim TW, Jun S, Ha Y, Yadav RK, Kumar A, Yoo C-Y, Oh I, Lim H-K, Shin JW, Ryoo R, Kim H, Kim J, Baeg J-O, Ihee H. 2019. Ultrafast charge transfer coupled with lattice phonons in two-dimensional covalent organic frameworks. Nat Commun 10:1873. doi:10.1038/s41467-019-09872-w. 31015440 PMC6478948

[66] Kim C, Kim TW, Kim S, Oh I, Wonneberger H, Yoon K, Kwak M, Kim J, Kim J, Li C, Müllen K, Ihee H. 2020. Molecular-level understanding of excited states of N-annulated rylene dye for dye-sensitized solar cells. J Phys Chem C 124:22993–23003. doi:10.1021/acs.jpcc.0c06532.

[67] Teramura M, Harada J, Tamiaki H. 2016. In vitro stereospecific hydration activities of the 3-vinyl group of chlorophyll derivatives by BchF and BchV enzymes involved in bacteriochlorophyll c biosynthesis of green sulfur bacteria. Photosynth Res 130:33–45. doi:10.1007/s11120-016-0220-7. 26816140

[68] Lange C, Kiesel S, Peters S, Virus S, Scheer H, Jahn D, Moser J. 2015. Broadened substrate specificity of 3-hydroxyethyl bacteriochlorophyllide a dehydrogenase (BchC) indicates a new route for the biosynthesis of bacteriochlorophyll a. J Biol Chem 290:19697–19709. doi:10.1074/jbc.M115.660555. 26088139 PMC4528133

