# Supplementary material for: The Photoactive Photosynthetic Reaction Center of a Rhodobacter sphaeroides Mutant Lacking 3-Vinyl (Bacterio)Chlorophyllide a Hydratase Contains 3-Vinyl Bacteriochlorophyll a
Source: Microbiol Spectr. 2023 Mar 27;11(2):e03878-22. doi: 10.1128/spectrum.03878-22 (PMC10101016; doi:10.1128/spectrum.03878-22)
Supplement: Supplemental file 1 — Supplemental material. Download spectrum.03878-22-s0001.pdf, PDF file, 1.3 MB [file spectrum.03878-22-s0001.pdf]

Supplemental Figure 1

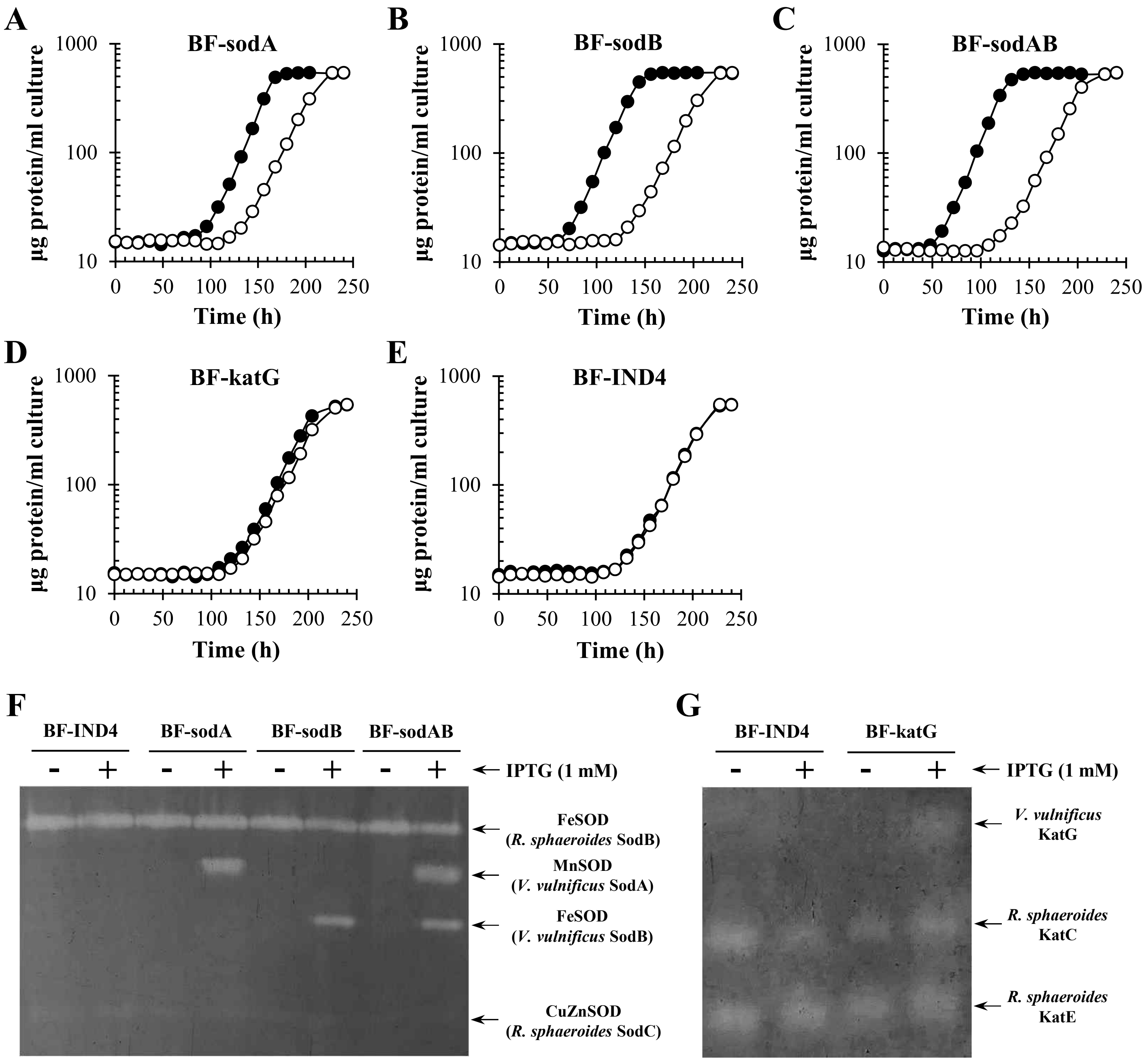

Supplemental Figure 1

Figure S1. Effect of SodA, SodB, and KatG on the photoheterotrophic growth of BF.

Photoheterotrophic growth of BF expressing *V. vulnificus sodA* (BF-sodA, A), *V. vulnificus sodB (*BF-sodB, B), *V. vulnificus sodA* and *sodB* (BF-sodAB, C), and *V. vulnificus katG* (BF-katG, D) (Table S1) were recorded with total protein of cells cultured without 10 mM DMSO in the presence (closed circles) or absence (open circles) of 1 mM IPTG. BF carrying empty vector (BF-pIND, E) was included as a control. Cellular SOD (F) and catalase (G) activities were visualized by in-gel activity staining of native polyacrylamide (10%) gel loaded with the lysates of cells harvested at exponential phase.

Supplemental Figure 2

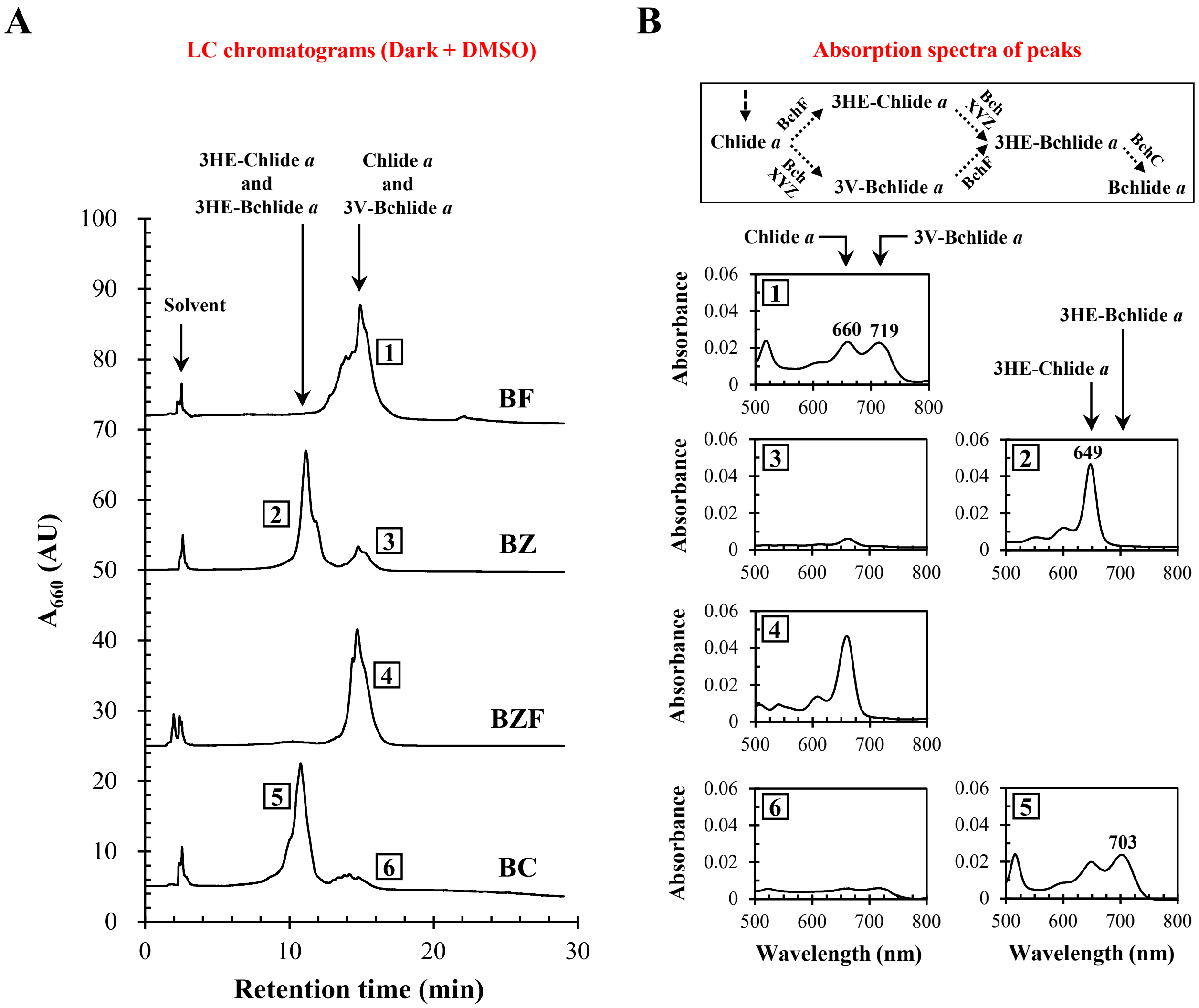

Supplemental Figure 2

Figure S2. Pigment analysis of mutants deficient in Bchl *a*-synthesis genes.

BF, BZ, BZF, and BC (Table S1) were cultured for 7 d without light (dark) in the presence of 75 mM DMSO as a terminal electron acceptor. Pigments were extracted from total cell lysates using extraction solution (acetone:methanol = 7:2, v/v), and analyzed by HPLC (A). Chlide *a*, 3HE-Chlide *a*, 3V-Bchlide *a*, and 3HE-Bchlide *a* were illustrated in metabolic pathway on top of the right panel (B). The absorption spectra (B) were shown to the right of the corresponding HPLC chromatograms (A) with pigment names on the peaks (B).

Supplemental Figure 3

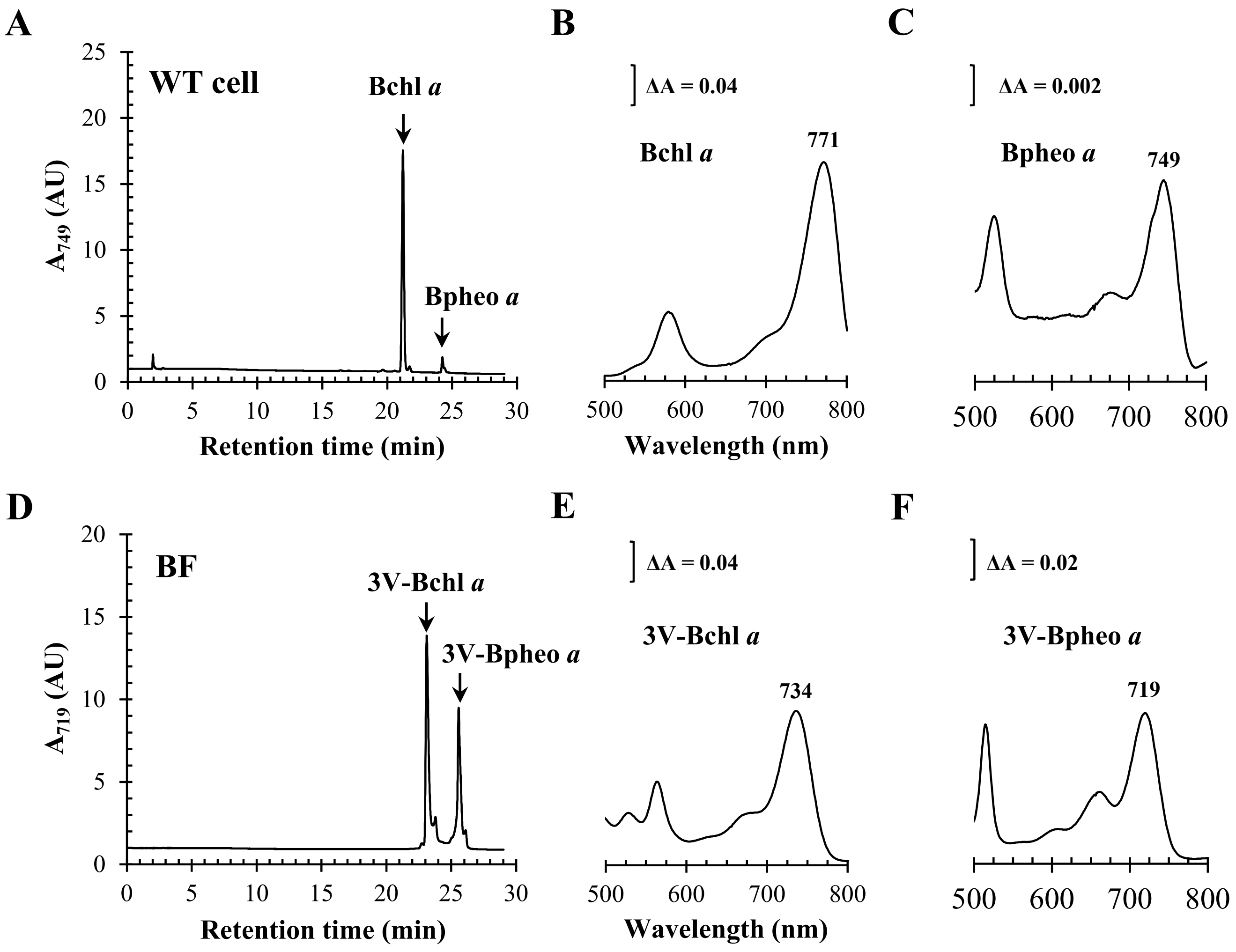

Supplemental Figure 3

Figure S3. Analysis of Bpheo *a* from WT cell and 3V-Bpheo *a* from BF.

Phytylated pigments were extracted from the membranes of WT (A) and BF cells (D) grown photoheterotrophically with 10 mM DMSO, and analyzed by HPLC. Bpheo *a* from WT membrane was monitored through absorbance at 749 nm (A), whereas 3V-Bpheo *a* was detected at 719 nm (D). Bchl *a* (A) and 3V-Bchl *a* (D) were also illustrated in the chromatograms. Bchl *a* (B), Bpheo *a* (C), 3V-Bchl *a* (E), and 3V-Bpheo *a* (F) were confirmed by measuring the absorption spectra.

Supplemental Figure 4

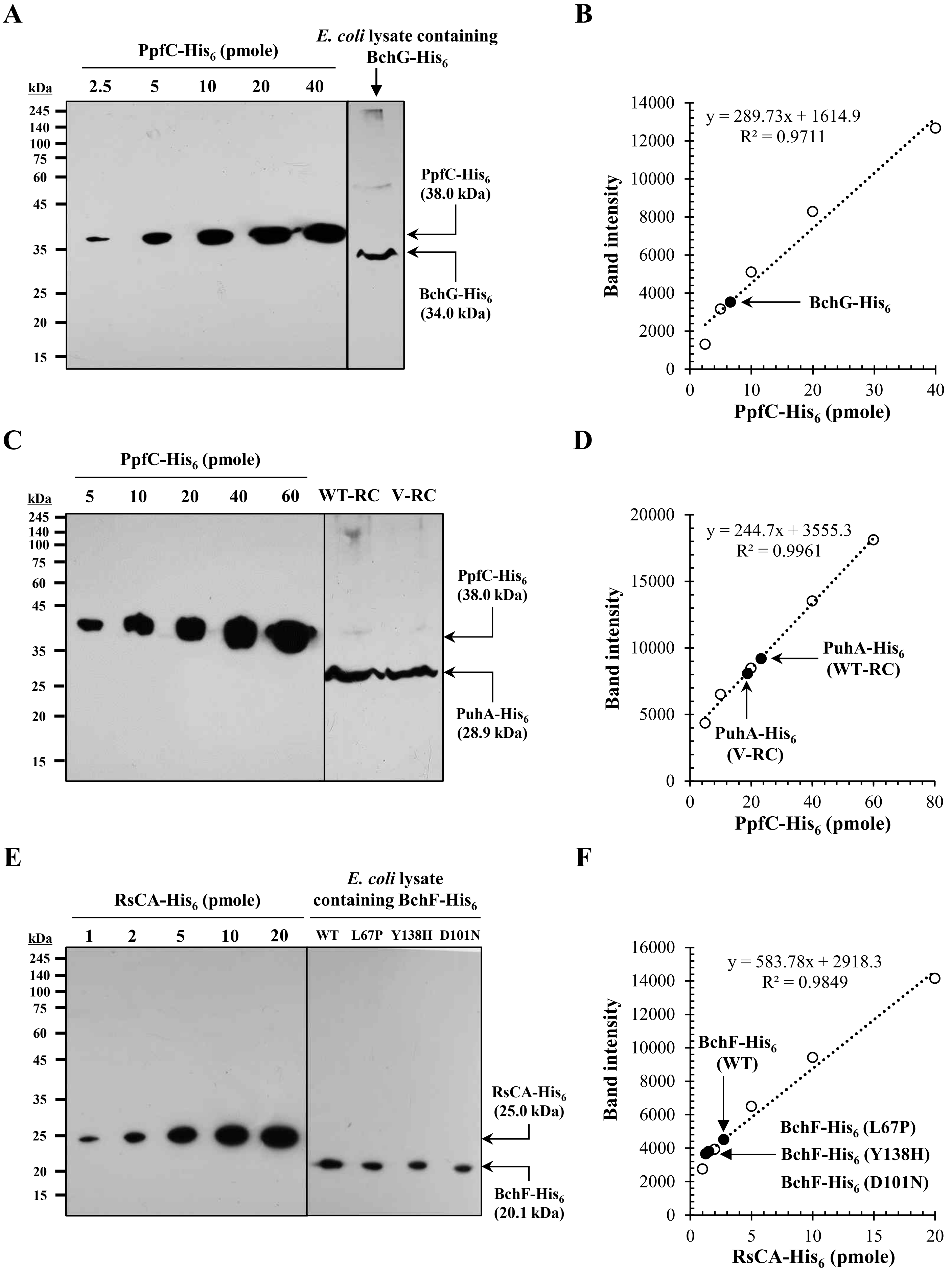

Supplemental Figure 4

Figure S4. Quantification of His_6-tagged proteins via western immunoblot using anti-His_6-tag antibody. BchG with C-terminal His_6-tag was produced in *E. coli* BL-Rsb (Table S1), and the cell lysate was subjected to western immunoblot analysis using an anti-His_6-tag antibody to determine the BchG level (A). Varying amount of protoporphyrin ferrochelatase of *Vibrio vulnificus* with C-terminal His_6-tag (PpfC-His_6) was used as a quantification standard (A). Band intensities were scanned by densitometer, and standard curve was constructed (B). BchG-His_6 in the lysate at 0.1 mg protein amounted to 6.6 pmole. In the same manner, C-terminally His_6-tagged subunit-H (PuhA-His_6) of the purified WT-RC and V-RC was quantified (C). PpfC-His_6 was used as a quantification standard (D). PuhA-His_6 in 2.5 μg WT-RC and 2.0 μg V-RC was determined to be 23.3 and 18.8 pmole, respectively. BchF (WT), $BchF^{L67P}$ (L67P), $BchF^{Y138H}$ (Y138H), and $BchF^{D101N}$ (D101N) were produced in *E. coli* BL-WT, BL-L67P, BL-Y138H, and BL-D101N, respectively, and the cell lysates were subjected to western immunoblot analysis using an anti-His_6-tag antibody to determine the BchF protein levels (E and F). C-terminally His_6-tagged carbonic anhydrase of *R. sphaeroides* (RsCA-His_6) was used as a quantification standard. The amounts of BchF-His_6 in cell lysates at 0.02 mg protein were determined to be 2.7, 1.5, 1.5, and 1.3 pmole for WT, L67P, Y138H, and D101N, respectively.

Supplemental Figure 5

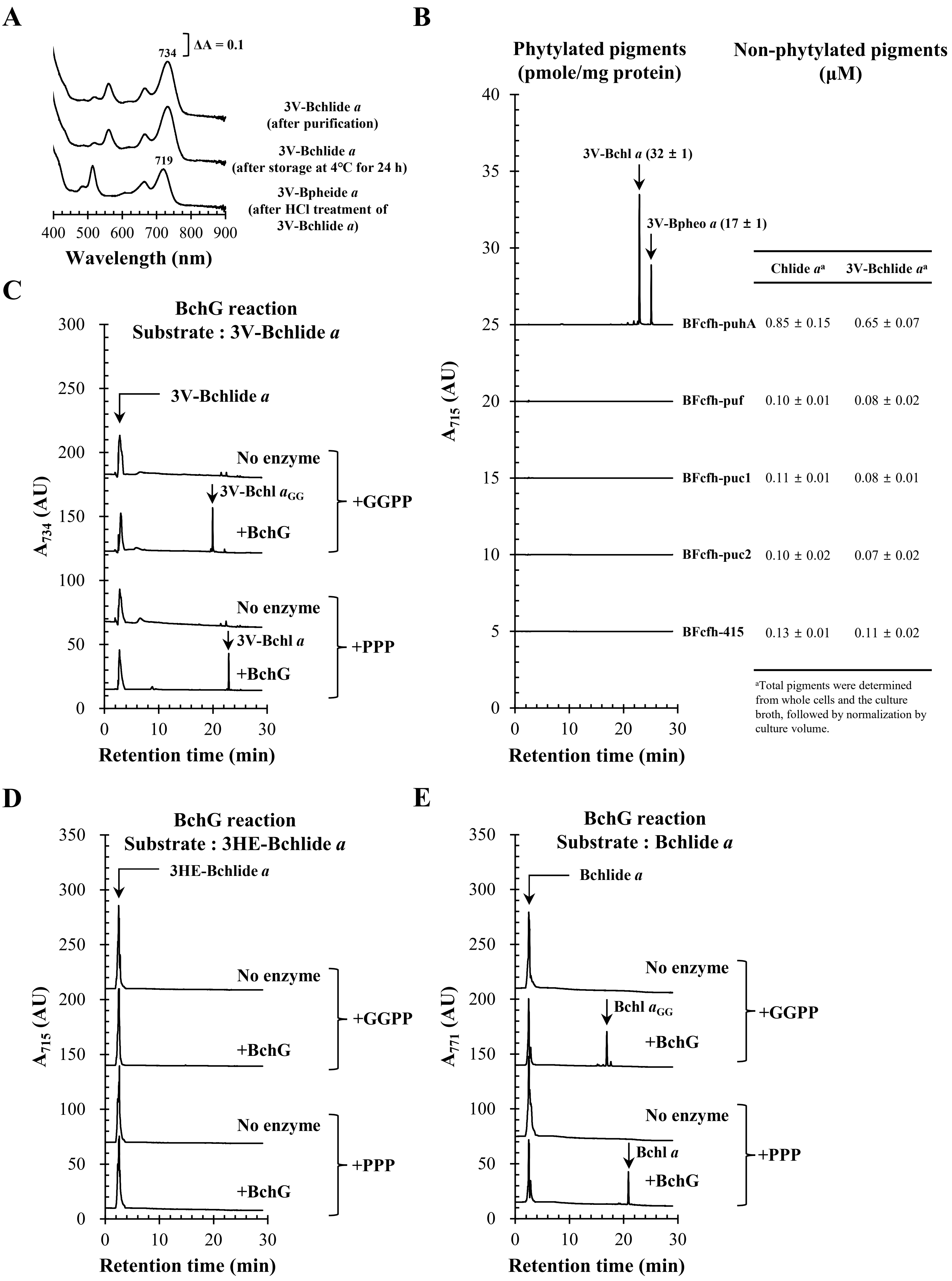

Supplemental Figure 5

Figure S5. BchG reaction with 3V-Bchlide *a* and 3HE-Bchlide *a*, and the pigment analysis of BFcfh recombinant strains grown under semi-aerobic conditions.

The stability of 3V-Bchlide *a* was examined via absorption spectral analysis (A). 3V-Bchlide *a* was prepared from 3V-Bchl *a* using AtChlase. It was subsequently treated with HCl (0.2 M) for 1 min to obtain Bpheide *a* (A), which was further examined after storage for 24 h at 4°C (A). BchG reaction with 3V-Bchlide *a* (C) and 3HE-Bchlide *a* (D) as the substrates were examined in the presence of either GGPP or PPP. Reaction with Bchlide *a* was included as a control (E). *E. coli* lysate containing BchG-His_6 (1 mg protein) was used as an enzyme source, and the prenylated products were detected by HPLC (C, D, and E). The recombinant strains of BFcfh-puhA, BFcfh-puf, BFcfh-puc1, BFcfh-puc2, and BFcfh-415 were grown under semi-aerobic conditions, and the pigments from the membranes were extracted and analyzed for phytylated pigments by HPLC (B). Chlide *a* and Bchlide *a* were also extracted from both whole cells and the culture broth, and their levels were shown in a table to the right of the corresponding HPLC chromatograms (B). AU, arbitrary unit.

Supplemental Figure 6

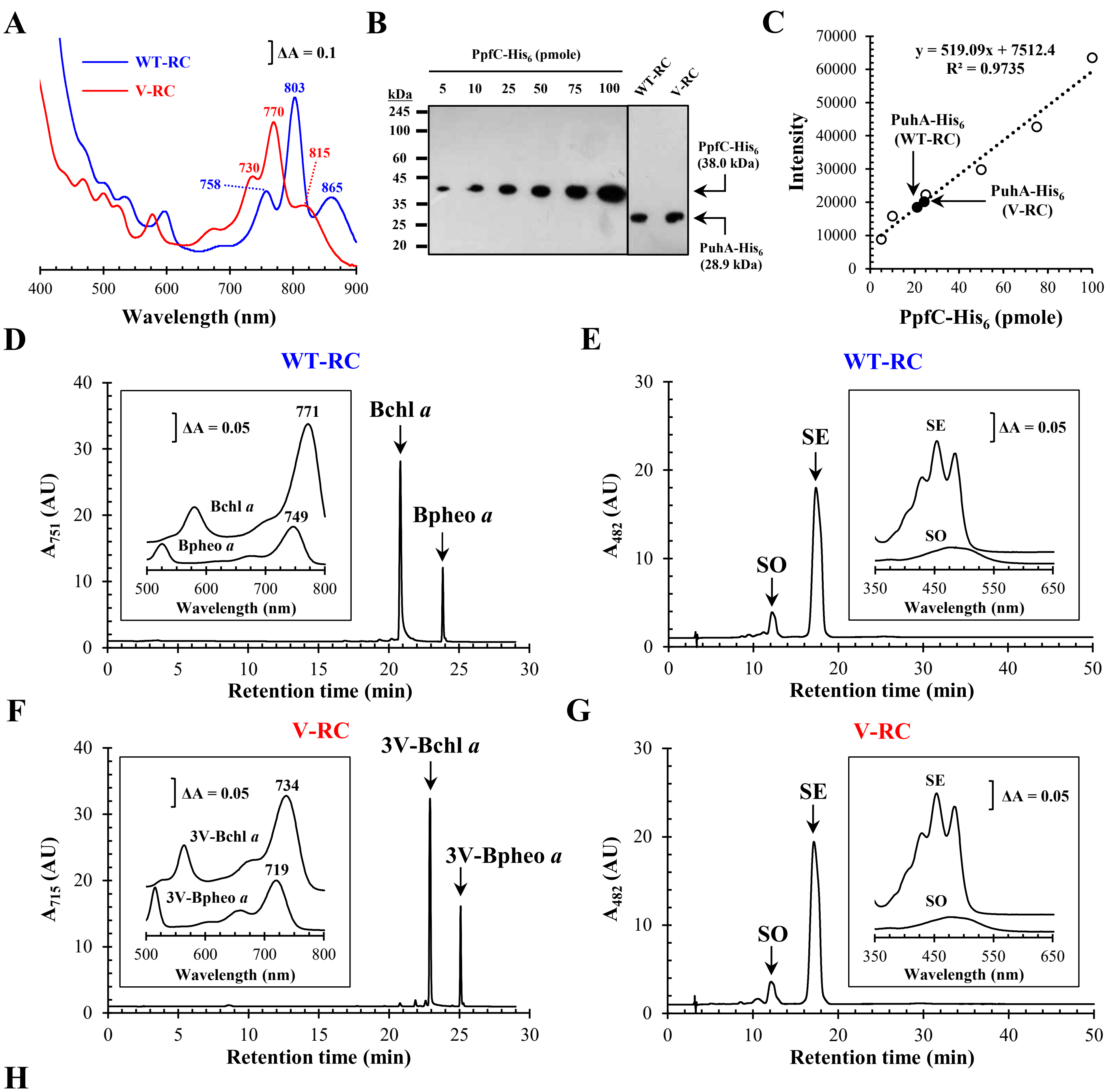

H

The number of pigment per RC

RC	Bchl *a*	3V-Bchl *a*	Bpheo *a*	3V-Bpheo *a*	SO	SE
**WT-RC**	4.17 ± 0.32	ND[a]	1.89 ± 0.19	ND	0.10 ± 0.01	0.80 ± 0.05
**V-RC**	ND	3.83 ± 0.09	ND	1.80 ± 0.06	0.11 ± 0.01	0.86 ± 0.07

[a]Not detected.

I

The pigment level and RC amount of BFcfh-puhA

3V-Bchl *a*[a] (pmole/mg)	3V-Bpheo *a*[a] (pmole/mg)	RC[b] (pmole/mg)
263 ± 6	118 ± 13	70 ± 3

[a]Total phytylated pigments were extracted from membrane, analyzed, and normalized by protein level (mg).
[b]Estimated by PuhA-His_6 level in membrane using western immunoblot.

Supplemental Figure 6

Figure S6. Analysis of WT-RC and V-RC purified from the cells grown photoheterotrophically in the presence of 10 mM DMSO.

The WT-RC and V-RC were purified from Wcfh-puhA and BFcfh-puhA, respectively, cultured photoheterotrophically with 10 mM DMSO, and the absorption spectra were recorded with λ_{max} of Q_y peaks on the peaks (A). Both RCs were quantified by western immunoblot analysis using PpfC-His_6 as the protein standard (B) and densitometric scan (C), by which 21.1 and 24.8 pmole of PuhA-His_6 were detected with 2 μg of the WT-RC and V-RC, respectively. The pigments were extracted from 10 μg of the WT-RC (D and E) and V-RC (F and G) and subjected to HPLC analysis. Bchl *a* (with Bpheo *a*) of WT-RC (D), 3V-Bchl *a* (with 3V-Bpheo *a*) of V-RC (F), and their carotenoids (E and G) were quantified. Bchl *a* and Bpheo *a* of WT-RC were monitored at 751 nm, where both pigments have the same molar extinction coefficient (D). Likewise, 3V-Bchl *a* and 3V-Bpheo of V-RC pigments were scanned at 715 nm (F). Carotenoids were monitored at 482 nm (E and G). Absorption spectrum of each pigment was illustrated with λ_{max} of the prominent peaks on the insets of D, E, F, and G. Collectively, the pigment contents per RC were determined (H), and shown with mean ± standard deviation (SD). BFcfh-puhA was grown photoheterotrophically with 10 mM DMSO, and the membranes were analyzed to determine the levels of phytylated pigments and RC (I). AU, arbitrary unit.

Supplemental Figure 7

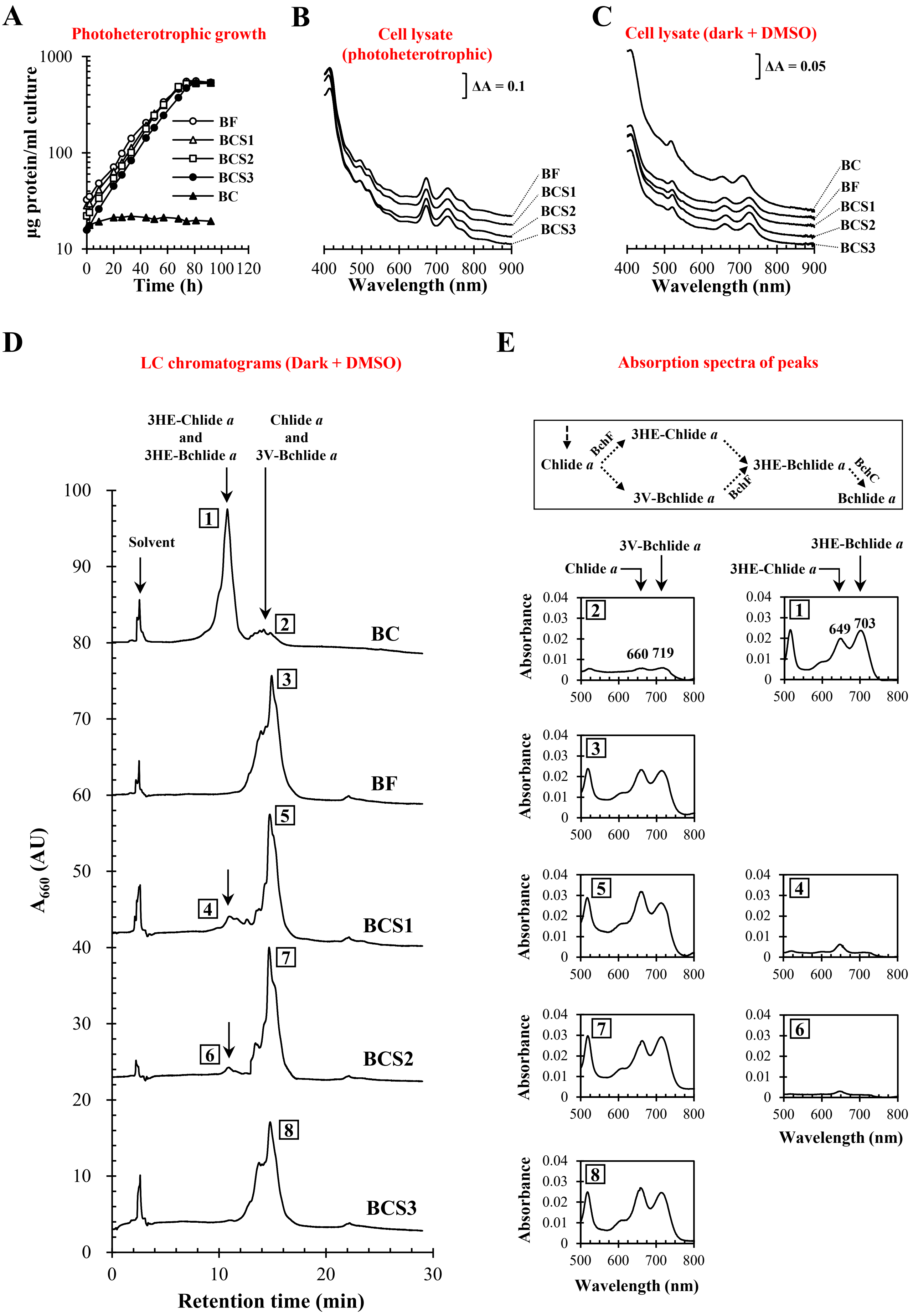

Supplemental Figure 7

Figure S7. Pigment analysis of the suppressor strains of BC.

BC did not grow photoheterotrophically (A). However, prolonged incubation (~2 weeks) of BC under photoheterotrophic conditions resulted in the emergence of growing suppressor cells at a frequency of $\sim 10^{-8}$. Three suppressor strains of BC were randomly selected from three independent selection trials. DNA sequence analysis of the *bchF* region from the chromosome of three suppressors BCS1, BCS2, and BCS3 revealed the generation of point mutations, which were L67P, Y138H, and D101N, respectively. Photoheterotrophic growth of BC, BF, and the three BC suppressors were measured based on the total protein of cells grown in the presence of 10 mM DMSO as an antioxidant (A), and the absorption spectra of total cell lysates were recorded (B). Cells were grown in the dark with 75 mM DMSO as a terminal electron acceptor for 7 d, and the absorption spectra of total cellular lysates were recorded for comparative analysis of pigments (C). The pigments were extracted from total cell lysates, and analyzed by HPLC (D). Chlide *a*, 3HE-Chlide *a*, 3V-Bchlide *a*, and 3HE-Bchlide *a* were illustrated in metabolic pathway on top of the right panel (E). The absorption spectra (E) were shown to the right of the corresponding HPLC chromatograms (D) with pigment names on the peaks (E).

Supplemental Figure 8

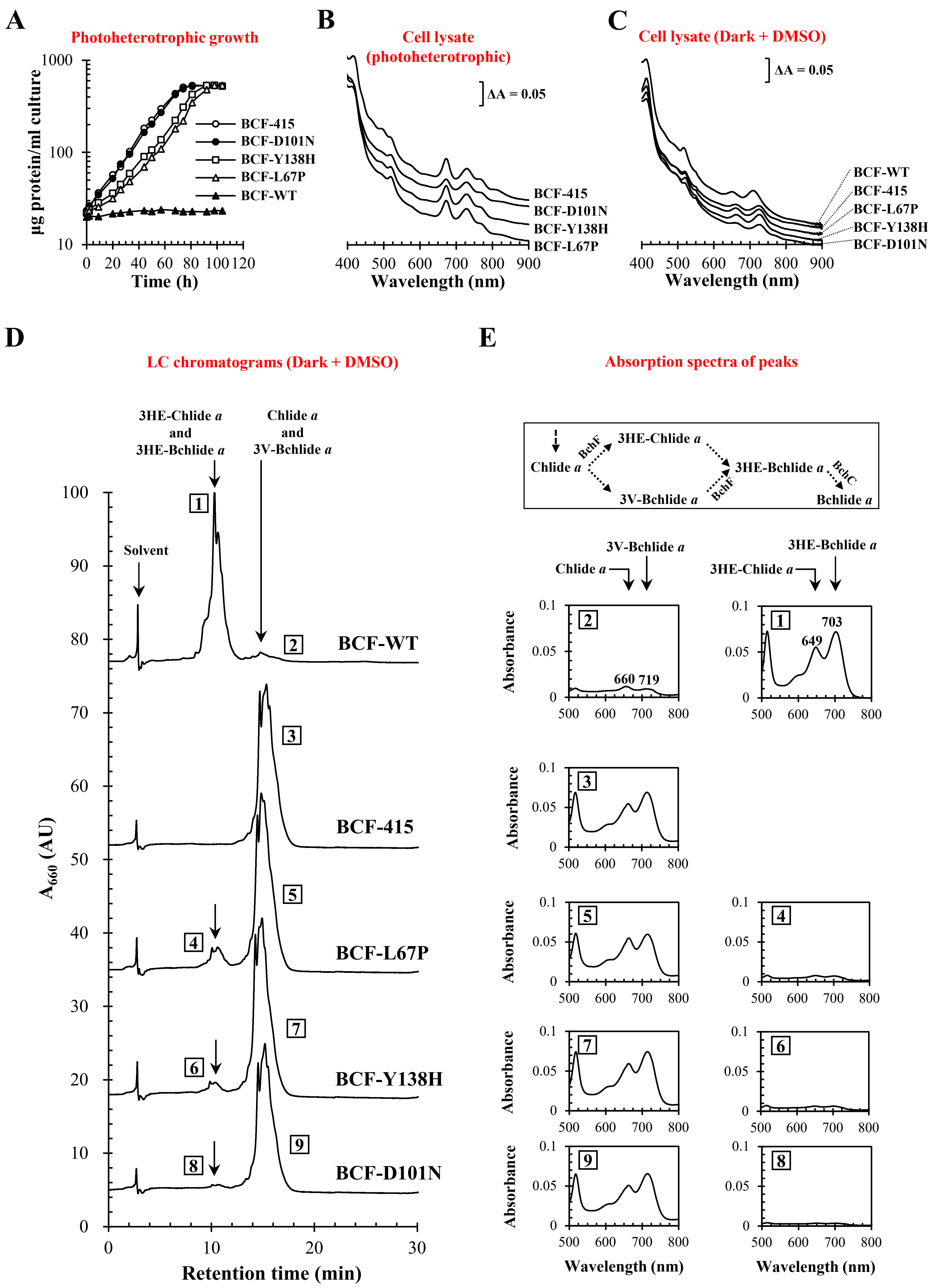

Supplemental Figure 8

Figure S8. Pigment analysis of BCF expressing the WT and mutated *bchF* DNAs.

Photoheterotrophic growth of BCF carrying pRK-BchF (BCF-WT), pRK-BchF^{L67P} (BCF-L67P), pRK-BchF^{Y138H} (BCF-Y138H), and pRK-BchF^{D101N} (BCF-D101N) (Table S1) were measured based on the total protein of cells grown in the presence of 10 mM DMSO as an antioxidant (A), and the absorption spectra of total cell lysates were recorded (B). BCF carrying an empty vector (BCF-415) was included as a control. Cells were grown in the dark with 75 mM DMSO as a terminal electron acceptor for 7 d, and the absorption spectra of total cellular lysates were recorded for comparative analysis of pigments (C). The pigments were extracted from total cell lysates, and analyzed by HPLC (D). Chlide *a*, 3HE-Chlide *a*, 3V-Bchlide *a*, and 3HE-Bchlide *a* were illustrated in metabolic pathway on top of the right panel (E). The absorption spectra (E) were shown to the right of the corresponding HPLC chromatograms (D) with pigment names on the peaks (E).

Supplemental Table 1

Table S1. Bacterial strains, primers and plasmids

Strains	Relevant characteristics	Source of reference
R. sphaeroides		
R. sphaeroides 2.4.1	Type strain (WT)	(S1)
BZ	Km^r; Previously known as BZ1; Δ*bchZ* mutant; interruption by Km^r DNA	(S2)
BZF	Km^r; Previously known as BZF1; Δ*bchZ* Δ*bchF* mutant; BZ with internal deletion in *bchF*	(S2)
BF	Δ*bchF* mutant; internal deletion in *bchF*	This study
BC	Δ*bchC* mutant; internal deletion in *bchC*	This study
BP	Δ*bchP* mutant; WT with internal deletion in *bchP*	This study
BFP	Δ*bchF* Δ*bchP* mutant; BF with internal deletion in *bchP*	This study
BCF	Δ*bchC* Δ*bchF* mutant; BC with internal deletion in *bchF*	This study
BFc	Δ*bchF* Δ*puc12BA* mutant; BF with internal deletion in *puc1BA* and *puc2BA*	This study
BFcf	Δ*bchF* Δ*puc12BA* Δ*pufBA* mutant; BFc with internal deletion in *pufBA*	This study
Wcfh	Δ*puc12BA* Δ*pufBA* Δ*puhA* mutant; WT with internal deletion in *puc1BA*, *puc2BA*, *pufBA*, and *puhA*	This study
BFcfh	Δ*bchF* Δ*puc12BA* Δ*pufBA* Δ*puhA* mutant; BFcf with internal deletion in *puhA*	This study
BCS1	Photoheterotrophically competent BC suppressor; BC with a mutation at the 67th residue of BchF (CTG → CCG)	This study
BCS2	Photoheterotrophically competent BC suppressor; BC with a mutation at the 138th residue of BchF (TAC → CAC)	This study
BCS3	Photoheterotrophically competent BC suppressor; BC with a mutation at the 101st residue of BchF (GAT → AAT)	This study
BF-sodA	Km^r; BF + pIND-sodA	This study
BF-sodB	Km^r; BF + pIND-sodB	This study
BF-sodAB	Km^r; BF + pIND-sodAB	This study
BF-katG	Km^r; BF + pIND-katG	This study
BF-IND4	Km^r; BF + pIND4	This study
Wcfh-puhA	Tc^r; Wcfh + pRK-PuhA, for purification of RC with C-terminally His$_6$-tagged PuhA	This study
Wcfh-puf	Tc^r; Wcfh + pRK-Puf, for purification of LH1 with C-terminally His$_6$-tagged PufA	This study
Wcfh-puc1	Tc^r; Wcfh + pRK-Puc1, for purification of LH2-1 with C-terminally His$_6$-tagged Puc1A	This study
Wcfh-puc2	Tc^r; Wcfh + pRK-Puc2, for purification of LH2-2 with C-terminally His$_6$-tagged Puc2A	This study
Wcfh-415	Tc^r; Wcfh + pRK415	This study
BFcfh-puhA	Tc^r; BFcfh + pRK-PuhA, for purification of RC with C-terminally His$_6$-tagged PuhA	This study
BFcfh-puf	Tc^r; BFcfh + pRK-Puf, for purification of LH1 with C-terminally His$_6$-tagged PufA	This study
BFcfh-puc1	Tc^r; BFcfh + pRK-Puc1, for purification of LH2-1 with C-terminally His$_6$-tagged Puc1A	This study
BFcfh-puc2	Tc^r; BFcfh + pRK-Puc2, for purification of LH2-2 with C-terminally His$_6$-tagged Puc2A	This study
BFcfh-415	Tc^r; BFcfh + pRK415	This study
BCF-WT	Tc^r; BCF + pRK-BchF	This study
BCF-L67P	Tc^r; BCF + pRK-BchF^{L67P}	This study
BCF-Y138H	Tc^r; BCF + pRK-BchF^{Y138H}	This study
BCF-D101N	Tc^r; BCF + pRK-BchF^{D101N}	This study
BCF-415	Tc^r; BCF + pRK415	This study
BFcf-WT	Tc^r; BFcf + pRK-BchF	This study
BFcf-L67P	Tc^r; BFcf + pRK-BchF^{L67P}	This study
BFcf-Y138H	Tc^r; BFcf + pRK-BchF^{Y138H}	This study
BFcf-D101N	Tc^r; BFcf + pRK-BchF^{D101N}	This study
BFcf-415	Tc^r; BFcf + pRK415	This study
E. coli		
DH5α *phe*	*sup*E44 Δ*lacU*169 (Φ80 *lacZ* ΔM15) *hsd*R17 *recA1 endA1 gyrA96 thi*-1 *rel*A1 *phe*::Tn*10d*Cm	(S3)
S17-1	C600::RP-4 2-(Tc::Mu)(Km::Tn7) *thi pro hsdR hsdM$^+$ recA*	(S4)
BL21 (DE3)	*E. coli* B F$^-$ *dcm ompT hsdS*(rB$^-$ mB$^-$) gal λ(DE3)	Stratagene
BL-Chlase	Ap^r; BL21 (DE3) + pChlase	This study
BL-Rsb	Km^r; BL21 (DE3) + pET-Rsb	This study
BL-WT	Km^r; BL21 (DE3) + pET-BchF	This study
BL-L67P	Km^r; BL21 (DE3) + pET-BchF^{L67P}	This study
BL-Y138H	Km^r; BL21 (DE3) + pET-BchF^{Y138H}	This study
BL-D101N	Km^r; BL21 (DE3) + pET-BchF^{D101N}	This study
BL-BchC	Km^r; BL21 (DE3) + pET-BchC	This study
BL-RsCA	Km^r; BL21 (DE3) + pET-RsCA	This study

Supplemental Table 1

Table S1. Bacterial strains, primers and plasmids

Primers	Nucleotide sequence (from 5' end to 3' end)	Note
B1F	GCATGCTCGGCCGCGAAGTGAGAG	*Sph*I is underlined.
B1R	GAATTCCGCATCGCG TCGCGCAC	*Eco*RI is underlined.
B2F	GAATTCCTTGCGGCCTATGCGACC	*Eco*RI is underlined.
B2R	TCTAGAGGCAGCGTCG GCACGATG	*Xba*I is underlined.
B3F	GCATGCCCGCACCGCGAGACAGAC	*Sph*I is underlined.
B3R	GAATTCCATCTGCCCGGTGTAGAA	*Eco*RI is underlined.
B4F	GAATTCTCACCCACACCCGACCGG	*Eco*RI is underlined.
B4R	TCTAGATCCCAGTCGTGGAAGCCG	*Xba*I is underlined.
B5F	GCATGCCGCTGATCGGCCAGATGC	*Sph*I is underlined.
B5R	GAATTCCACGATCTGGCTGTCGGG	*Eco*RI is underlined.
B6F	GAATTCGTTCTCCGCTCGATGCAG	*Eco*RI is underlined.
B6R	TCTAGAACGCGGTCGGCGAAGACG	*Xba*I is underlined.
B7F	GCATGCGGCCGCCAAGCCATCCTG	*Sph*I is underlined.
B7R	GAATTCGCTCGGCCAGACTTTGTT	*Eco*RI is underlined.
B8F	GAATTCGTCGCGGCCGAGTAATGC	*Eco*RI is underlined.
B8R	TCTAGAAGCGCGAAGGGCATGATG	*Xba*I is underlined.
B9F	GCATGCGGGCACCTACGAGCTCAC	*Sph*I is underlined.
B9R	CTGCAGATCATCGGTCACTTGTAC	*Pst*I is underlined.
B10F	CTGCAGGTGACCGGCCTGCCGTTC	*Pst*I is underlined.
B10R	TCTAGACTCGTCGCCGTCGAGCTC	*Xba*I is underlined.
B11F	GCATGCTCGGGACGGAGATGGACAGC	*Sph*I is underlined.
B11R	GAATTCGTCCGTAAGACCTGTGTAG	*Eco*RI is underlined.
B12F	GAATTCATCCTGCTGAGCACCCCC	*Eco*RI is underlined.
B12R	TCTAGAGTAGGTGTAGCCCGTGTTCG	*Xba*I is underlined.
B13F	GCATGCCTGCTCGTGCTTTTCGC	*Sph*I is underlined.
B13R	GAATTCCGACGCCAGATCGAAGTT	*Eco*RI is underlined.
B14F	GAATTCGCGATGCTGGCCGAATAC	*Eco*RI is underlined.
B14R	TCTAGATGAAGGGCAGGTTCAGCG	*Xba*I is underlined.
B15F	GGATCCATGTCACACACTTTCCCTG	*Bam*HI is underlined.
B15R	AAGCTTTTAGCCTATTGCTTGAGCAT	*Hin*dIII is underlined.
B16F	GGATCCATGGCATTTGAACTACCAG	*Bam*HI is underlined.
B16R	AAGCTTTTATTTCGCT AGGTTCTCTGC	*Hin*dIII is underlined.
B17F	GGATCCATGGAACACAAACCTACCC	*Bam*HI is underlined.
B17R	AAGCTTTTAGATATCGAAGCGATCCG	*Hin*dIII is underlined.
B18F	GGTACCAACCGAAGGTAATCCCTTC	*Kpn*I is underlined.
B18R	GGTACCTTAGCCTATT GCTTGAGCAT	*Kpn*I is underlined.
B19F	GGTCTCGAATGGCGGCGATAGAGGAC	*Bsa*I is underlined.
B19R	GGTCTCAGCGCTGACGAAGATACCAGAAGCTTC	*Bsa*I is underlined.
B20F	CATATGAGTGTCAATCTATCCTTAC	*Nde*I is underlined.
B20R	AAGCTTCGGCAGCACCTCCAGCCC	*Hin*dIII is underlined.
B21F	CTGCAGCACGCCCTGAATGTGGGC	*Pst*I is underlined.
B21R	TCTAGAGTCGGCCTGCACGCGGGC	*Xba*I is underlined.
B22F	TCTAGAACCTGACACCGGAGGACC	*Xba*I is underlined.
B22R	GAATTCTCA**GTGGTGGTGGTGGTGGTG**GGCGTATTCGGCCAGCATCGC	*Eco*RI is underlined, His$_6$-tag sequence is in boldface.
B23F	TCTAGACGCCACCGCAGGCTTCCC	*Xba*I is underlined.
B23R	GGTACCTTA**GTGGTGGTGGTGGTGGTG**CCCGCCCTCGGCGACGGCGACGCG	*Kpn*I is underlined, His$_6$-tag sequence is in boldface.
B24F	AAGCTTACGCCCTGAATGTGGGCG	*Hin*dIII is underlined.
B24R	TCTAGATTA**GTGGTGGTGGTGGTGGTG**CCCGCCCTCGGCCGCGACCGCAGC	*Xba*I is underlined, His$_6$-tag sequence is in boldface.
B25F	TCTAGAGGCCCTACACGCATCGAG	*Xba*I is underlined.
B25R	GGTACCTTA**GTGGTGGTGGTGGTGGTG**CCCGCCTTGCGCGGCCGGAACGAA	*Kpn*I is underlined, His$_6$-tag sequence is in boldface.
B26F	CATATGCAGCCCACGTCCCCCGCAG	*Nde*I is underlined.
B26R	AAGCTTTGCGCGGCCTCCATGTC	*Hin*dIII is underlined.
B27F	AAGCTTATCACCAGCTCCGCCGGG	*Hin*dIII is underlined.
B27R	GGTACCTCATTGCGCG GCCTCCATG	*Kpn*I is underlined.
B28F	CATATGAGAACGACCGCCGTCATC	*Nde*I is underlined.
B28R	AAGCTTTGCGGTG GCCCTCCAATC	*Hin*dIII is underlined.
B29F	CATATGCACAATGCGAGGCCGC	*Nde*I is underlined.
B29R	AAGCTTGACCGGCACGAAGCCCTG	*Hin*dIII is underlined.

Supplemental Table 1

Table S1. Bacterial strains, primers and plasmids

Plasmids	Relevant characteristics	Source of reference
pLO1	Km^{r}; *sacB*$^{+}$, RP4 *oriT*, ColE1 *ori,* suicide vector for mutant construction	(S5)
pIND4	Km^{r}; *lacI*^{q}, pMG160 *oriT*, ColE1 *ori,* inducible expression vector for His$_6$-tagged protein in *R. sphaeroides*	(S6)
pASK-IBA3plus	Ap^{r}, inducible expression vector for strep-tagged protein in *E. coli*	IBA Life sciences
pET29a	Km^{r}, inducible expression vector for His$_6$-tagged protein in *E. coli*	Novagen
pRK415	Tc^{r}; *ori* IncP Mob RP4 *lacZα*, expression vector for *R. sphaeroides*	(S7)
pLO-bchF	pLO1 + 1,114-bp *Sph*I/*Xba*I fragment containing internally deleted *bchF* gene	This study
pLO-bchC	pLO1 + 1,044-bp *Sph*I/*Xba*I fragment containing internally deleted *bchC* gene	This study
pLO-bchP	pLO1 + 1,046-bp *Sph*I/*Xba*I fragment containing internally deleted *bchP* gene	This study
pLO-puc1BA	pLO1 + 1,012-bp *Sph*I/*Xba*I fragment containing internally deleted *puc1BA* gene	This study
pLO-puc2BA	pLO1 + 967-bp *Sph*I/*Xba*I fragment containing internally deleted *puc2BA* gene	This study
pLO-pufBA	pLO1 + 1,355-bp *Sph*I/*Xba*I fragment containing internally deleted *pufBA* gene	This study
pLO-puhA	pLO1 + 1,010-bp *Sph*I/*Xba*I fragment containing internally deleted *puhA* gene	This study
pIND-sodA	pIND4 + 609-bp *Bam*HI/*Hin*dIII fragment containing *V. vulnificus sodA* gene	This study
pIND-sodB	pIND4 + 585-bp *Bam*HI/*Hin*dIII fragment containing *V. vulnificus sodB* gene	This study
pIND-sodAB	pIND4 + 1,234-bp *Bam*HI/*Hin*dIII fragment containing *V. vulnificus sodA* and *sodB* genes	This study
pIND-katG	pIND4 + 2,172-bp *Bam*HI/*Hin*dIII fragment containing *V. vulnificus katG* gene	This study
pChlase	pASK-IBA3plus + 972-bp *Bsa*I fragment containing *A. thaliana* chlorophyllase gene	This study
pET-Rsb	pET29a + 906-bp *Nde*I/*Hin*dIII fragment containing *R. sphaeroides bchG* gene	This study
pRK-PuhA	pRK415 + 1,499-bp *Pst*I/*Eco*RI fragment containing *puc* promoter and *puhA* gene with C-terminal His$_6$ codon, for expression of His$_6$-tagged RC in *R. sphaeroides*	This study
pRK-Puf	pRK415 + 1,252-bp *Pst*I/*Kpn*I fragment containing *puc* promoter and *pufBA* gene with C-terminal His$_6$ on *pufA*, for expression of His$_6$-tagged LH1 in *R. sphaeroides*	This study
pRK-Puc1	pRK415 + 1,068-bp *Hin*dIII/*Xba*I fragment containing *puc* promoter and *puc1BA* gene with C-terminal His$_6$ on *puc1A*, for expression of His$_6$-tagged LH2-1 in *R. sphaeroides*	This study
pRK-Puc2	pRK415 + 1,697-bp *Pst*I/*Kpn*I fragment containing *puc* promoter and *puc2BA* gene with C-terminal His$_6$ on *puc2A*, for expression of His$_6$-tagged LH2-2 in *R. sphaeroides*	This study
pET-BchF	pET29a + 480-bp *Nde*I/*Hin*dIII fragment containing WT *bchF* gene	This study
pET-BchF^{L67P}	pET-BchF with point mutation of L67P (CTG → CCG)	This study
pET-BchF^{Y138H}	pET-BchF with point mutation of Y138H (TAC → CAC)	This study
pET-BchF^{D101N}	pET-BchF with point mutation of D101N (GAT → AAT)	This study
pRK-BchF	pRK415 + 857-bp *Hin*dIII/*Kpn*I fragment containing WT *bchF* gene	This study
pRK-BchF^{L67P}	pRK-BchF with point mutation of L67P (CTG → CCG) on *bchF*	This study
pRK-BchF^{Y138H}	pRK-BchF with point mutation of Y138H (TAC → CAC) on *bchF*	This study
pRK-BchF^{D101N}	pRK-BchF with point mutation of D101N (GAT → AAT) on *bchF*	This study
pET-BchC	pET29a + 954-bp *Nde*I/*Hin*dIII fragment containing *R. sphaeroides bchC* gene	This study
pET-RsCA	pET29a + 642-bp *Nde*I/*Hin*dIII fragment containing *R. sphaeroides* carbonic anhydrase	This study

Supplemental Text 1

Plasmid constructions

1. Plasmid for in-frame deletion of *bchF*

The 522-bp DNA upstream from the 24th codon and the 592-bp DNA downstream from the 132nd codon of *Rhodobacter sphaeroides bchF* (RSP_0284) were PCR-amplified using the primer sets of B1F/B1R and B2F/B2R, respectively (all primers are listed in Table S1). The DNA fragment from B1F/B1R was digested with *Sph*I/*Eco*RI, and the fragment from B2F/B2R was digested with *Eco*RI/*Xba*I. The resulting fragments were ligated into *Sph*I/*Xba*I sites of pLO1 (S5) to yield pLO-bchF.

2. Plasmid for in-frame deletion of *bchC*

The 494-bp DNA upstream from the 51st codon and the 550-bp DNA downstream from the 286th codon of *R. sphaeroides bchC* (RSP_0263) were PCR-amplified using the primer sets of B3F/B3R and B4F/B4R, respectively. The fragments were digested with the restriction enzymes and ligated into pLO1 in the same manner as pLO-bchF to yield pLO-bchC.

3. Plasmid for in-frame deletion of *bchP*

A 547-bp DNA upstream from the 60th codon and the 499-bp DNA downstream from the 326th codon of *R. sphaeroides bchP* (RSP_0277) were PCR-amplified using the primer sets of B5F/B5R and B6F/B6R, respectively. The fragments were digested with the restriction enzymes and ligated into pLO1 in the same manner as pLO-bchF to yield pLO-bchP.

4. Plasmid for in-frame deletion of *puc1BA*

The 441-bp DNA upstream from the 11th codon of *R. sphaeroides puc1B* (RSP_0314) and the 571-bp DNA downstream from the 51st codon of *puc1A* (RSP_6256) were PCR-amplified using the primer sets of B7F/B7R and B8F/B8R, respectively. The fragments were digested with the restriction enzymes and ligated into pLO1 in the same manner as pLO-bchF to yield pLO-puc1BA.

5. Plasmid for in-frame deletion of *puc2BA*

The 436-bp DNA upstream from the 4th codon of *R. sphaeroides puc2B* (RSP_1556) and the 531-bp DNA downstream from the 253rd codon of *puc2A* (RSP_1557) were PCR-amplified using the primer sets of B9F/B9R and B10F/B10R, respectively. The fragments were digested with the restriction enzymes and ligated into pLO1 in the same manner as pLO-bchF to yield pLO-puc2BA.

6. Plasmid for in-frame deletion of *pufBA*

The 659-bp DNA upstream from the 14th codon of *R. sphaeroides pufB* (RSP_6108) and the 696-bp DNA downstream from the 34th codon of *pufA* (RSP_0258) were PCR-amplified using the primer sets of B11F/B11R and B12F/B12R, respectively. The fragments were digested with the restriction enzymes and ligated into pLO1 in the same manner as pLO-bchF to yield pLO-pufBA.

7. Plasmid for in-frame deletion of *puhA*

The 489-bp DNA upstream from the 14th codon and the 521-bp DNA downstream from the 254th codon of *R. sphaeroides puhA* (RSP_0291) were PCR-amplified using the primer sets of B13F/B13R and B14F/B14R, respectively. The fragments were digested with the restriction enzymes and ligated into pLO1 in the same manner as pLO-bchF to yield pLO-puhA.

8. Plasmids for inducible expression of SODs and catalase in *R. sphaeroides*

The structural gene of *Vibrio vulnificus sodA* (VVMO6_RS01095), *sodB* (VVMO6_RS10185), and *katG* (VVMO6_RS08565) were PCR-amplified using the primer sets of B15F/B15R, B16F/B16R, and B17F/B17R, respectively. The resulting 609-bp, 585-bp, and 2172-bp DNA fragments of *sodA*, *sodB*, and *katG* were digested with *Bam*HI/*Hin*dIII and ligated into pIND4 (S6) to construct pIND-sodA, pIND-sodB, and pIND-katG, respectively. For construction of pIND-sodAB, a 609-bp fragment of *sodA* was PCR-amplified using the primer set of B15F/B18R, and a 625-bp fragment of *sodB* extending from the 40 bp upstream of the start codon to its stop codon was PCR-amplified using the primer set of B18F/B16R. The *sodA* and *sodB* fragment were digested with *Bam*HI/*Kpn*I and *Kpn*I/*Hin*dIII, respectively, and the two fragments were ligated into *Bam*HI/*Hin*dIII site of pIND4 to construct pIND-sodAB.

Supplemental Text 1

Plasmid constructions

9. Plasmid for purification of *Arabidopsis thaliana* chlorophyllase

The gene fragment of chlorophyllase 1 (AtCLH1 as AtChlase) extending from the start codon to its penultimate codon was amplified by PCR from total cDNA of *A. thaliana* using the primer set of B19F/B19R. The 972-bp fragment was digested with *Bsa*I and ligated into pASK-IBA3plus (IBA Life sciences, Göttingen, Germany) to yield pChlase.

10. Plasmid for overexpression of BchG of *R. sphaeroides* in *E. coil*

The structural gene of *R. sphaeroides bchG* (RSP_0279) extending from the start codon to its penultimate codon was PCR-amplified using the primer set of B20F/B20R. The 906-bp PCR product was digested with *Nde*I/*Hin*dIII and cloned into pET29a (Novagen, Madison, WI, USA) to yield pET-Rsb.

11. Plasmids for purification of His_6-tagged RC and LHs from *R. sphaeroides*

The 698-bp fragment containing promoter region of *R. sphaeroides puc* operon (P_{puc}) (S8) was PCR-amplified using the primer set of B21F/B21R and digested with *Pst*I/*Xba*I. The 801-bp fragment from the 24 bp upstream of the start codon of *R. sphaeroides puhA* to its penultimate codon was PCR-amplified using the primer set of B22F/B22R, followed by digestion with *Xba*I/*Eco*RI and cloned into *Pst*I/*Eco*RI site of pRK415 (S7) together with P_{puc}, yielding pRK-PuhA. The 554-bp fragment from the 217 bp upstream of the start codon of *R. sphaeroides pufB* to the penultimate codon of *pufA* was PCR-amplified using the primer set of B23F/B23R, followed by digestion with *Xba*I/*Kpn*I and cloned into *Pst*I/*Kpn*I site of pRK415 together with P_{puc}, yielding pRK-Puf. The 1,068-bp fragment from the 735 bp upstream of the start codon of *R. sphaeroides puc1B* to the penultimate codon of *puc1A* was PCR-amplified using the primer set of B24F/B24R, digested with *Hin*dIII/*Xba*I, and cloned into the same site of pRK415, yielding pRK-Puc1. The 999-bp fragment from the 41 bp upstream of the start codon of *R. sphaeroides puc2B* to the penultimate codon of *puc2A* was PCR-amplified using the primer set of B25F/B25R, followed by digestion with *Xba*I/*Kpn*I and cloned into *Pst*I/*Kpn*I site of pRK415 together with P_{puc}, yielding pRK-Puc2.

12. Plasmids for overexpression of BchF of *R. sphaeroides* in *E. coil*

The structural gene of *R. sphaeroides bchF* (RSP_0284) extending from the start codon to its penultimate codon was PCR-amplified using the primer set of B26F/B26R. The 480-bp PCR product was digested with *Nde*I/*Hin*dIII and cloned into pET29a. Genomic DNA of WT cell, BCS1, BCS2, and BCS3 were used as templates for PCR using the primer set of B26F/B26R to yield pET-BchF, pET-$BchF^{L67P}$, pET-$BchF^{Y138H}$, and pET-$BchF^{D101N}$, respectively.

13. Plasmids for overexpression of BchF in *R. sphaeroides*

The gene fragment extending from the 374 bp upstream from the start codon of *R. sphaeroides bchF* (RSP_0284) to its stop codon was PCR-amplified using the primer set of B27F/B27R. The 857-bp PCR product was digested with *Hin*dIII/*Kpn*I and cloned into pRK415. Genomic DNA of WT cell, BCS1, BCS2, and BCS3 were used as templates for PCR using the primer set of B27F/B27R to yield pRK-BchF, pRK-$BchF^{L67P}$, pRK-$BchF^{Y138H}$, and pRK-$BchF^{D101N}$, respectively.

14. Plasmid for overexpression of BchC of *R. sphaeroides* in *E. coil*

The structural gene of *R. sphaeroides bchC* (RSP_0263) extending from the start codon to its penultimate codon was PCR-amplified using the primer set of B28F/B28R. The 954-bp PCR product was digested with *Nde*I/*Hin*dIII and cloned into pET29a to yield pET-BchC.

15. Plasmid for overexpression of carbonic anhydrase of *R. sphaeroides* in *E. coil*

The structural gene of *R. sphaeroides* carbonic anhydrase (RSP_1377, RsCA) extending from the start codon to its penultimate codon was PCR-amplified using the primer set of B29F/B29R. The 642-bp fragment was digested with *Nde*I/*Hin*dIII and cloned into pET29a to yield pET-RsCA.

Supplemental Text 1

Construction of *R. sphaeroides* mutants

Target genes were disrupted by in-frame deletion as described previously (S9). All plasmids for mutant construction (Table S1) were transformed into *E. coli* S17-1 (S4) and then mobilized into *R. sphaeoroides* through conjugation (S10). Single-crossover recombinants of *R. sphaeroides* with the Km^r phenotype were selected on Sis agar plate supplemented with Km, and subsequently segregated on medium containing 15% (w/v) sucrose to obtain double-crossover recombinants, Km^s and $sucrose^r$. The final mutants with internal gene deletions were confirmed by PCR analysis of genomic DNA.

R. sphaeroides mutants BF and BC were generated by mobilizing pLO-bchF and pLO-bchC, respectively, into WT cells. Mutants BP and BFP were generated by interrupting *bchP* in WT and BF cells, respectively, using pLO-bchP. Mutant BCF was generated by interrupting *bchF* in BC using pLO-bchF. Mutant BFc was generated by sequentially interrupting *puc1BA* and *puc2BA* in BF using pLO-puc1BA and pLO-puc2BA, respectively. Mutant BFcf was generated by interrupting *pufBA* from BFc using pLO-pufBA. Sequential interruption of *puc1BA*, *puc2BA*, *pufBA*, and *puhA* in WT cells using pLO-puc1BA, pLO-puc2BA, pLO-pufBA, and pLO-puhA, respectively, generated mutant Wcfh. Deletion of *puhA* from BFcf yielded BFcfh.

Spectral analysis of culture supernatant and cell membrane

R. sphaeroides cells showing exponential growth in the dark with 75 mM DMSO or under photoheterotrophic conditions with or without 10 mM DMSO were centrifuged at 6,000 × *g* for 5 min at 4°C and the supernatant was used for spectral analysis. The cell pellet was washed once with 10 mM phosphate-buffered saline (PBS, pH 7.4) and resuspended in the same buffer. Cells were disrupted by sonication (Branson Sonifier model 250; Danbury, CT, USA) on ice for 5 min, three times. Cell lysates were centrifuged at 6,000 × *g* for 10 min at 4°C and the supernatant was centrifuged at 100,000 × *g* for 1 h at 4°C. The membrane pellets were washed once with PBS and resuspended in PBS supplemented with 1% *n*-dodecyl β-D-maltoside (DDM), which was continuously mixed for 1 h at 4°C. Insoluble materials were removed by centrifugation at 12,000 × *g* for 5 min at 4°C. The membrane fraction in the supernatant was quantified by the Lowry method (S11), and the absorption spectrum of the sample (0.5 mg protein) was recorded using a Shimadzu UV 2550 spectrophotometer (Kyoto, Japan).

In-gel activity staining of SOD and catalase

Exponentially growing photoheterotrophic cells were harvested, washed, and resuspended in PBS. Cell lysates were obtained by sonication and loaded onto a native gel (10% polyacrylamide gel without sodium dodecyl sulfate [SDS] and dithiothreitol). SOD activity was visualized in a native gel using nitrotetrazolium blue (Sigma-Aldrich, St. Louis, MO, USA) as described previously (S12). Catalase activity was visualized in a native gel using H_2O_2, $FeCl_3$, and $K_3[Fe(CN)_6]$ as described previously (S13).

References for Supplemental materials

References

S1. Sistrom WR. 1960. A Requirement for Sodium in the Growth of *Rhodopseudomonas spheroides*. J Gen Microbiol 22:778–785.

S2. Kim E-J, Kim J-S, Lee I-H, Rhee HJ, Lee JK. 2008. Superoxide generation by chlorophyllide *a* reductase of *Rhodobacter sphaeroides*. J Biol Chem 283:3718–3730.

S3. Eraso JM, Kaplan S. 1994. *prrA*, a putative response regulator involved in oxygen regulation of photosynthesis gene expression in *Rhodobacter sphaeroides*. J Bacteriol 176:32–43.

S4. Simon R, Priefer U, Pühler A. 1983. A broad host range mobilization system for *in vivo* genetic engineering: transposon mutagenesis in gram negative bacteria. Nat Biotechnol 1:784–791.

S5. Lenz O, Schwartz E, Dernedde J, Eitinger M, Friedrich B. 1994. The *Alcaligenes eutrophus* H16 *hoxX* gene participates in hydrogenase regulation. J Bacteriol 176:4385–4393.

S6. Ind AC, Porter SL, Brown MT, Byles ED, de Beyer JA, Godfrey SA, Armitage JP. 2009. Inducible-expression plasmid for *Rhodobacter sphaeroides* and *Paracoccus denitrificans*. Appl Environ Microbiol 75:6613–6615.

S7. Keen NT, Tamaki S, Kobayashi D, Troilinger D. 1988. Improved brood-host-range plasmids for DNA cloning in gram-negative bacteria. Gene 70:191–197.

S8. Lee JK, Kaplan S. 1995. Transcriptional regulation of *puc* operon expression in *Rhodobacter sphaeroides*. J Biol Chem 270:20453–20458.

S9. Kim E-J, Kim J-S, Kim M-S, Lee JK. 2006. Effect of changes in the level of light harvesting complexes of *Rhodobacter sphaeroides* on the photoheterotrophic production of hydrogen. Int J Hydrogen Energy 31:531–538.

S10. Davis J, Donohue TJ, Kaplan S. 1988. Construction, characterization, and complementation of a Puf- mutant of *Rhodobacter sphaeroides*. J Bacteriol 170:320–329.

S11. Lowry OH, Rosebrough NJ, Farr AL, Randall RJ. 1951. Protein measurement with the Folin phenol reagent. J Biol Chem 193:265–275.

S12. Kho DH, Yoo S-B, Kim J-S, Kim E-J, Lee JK. 2004. Characterization of Cu- and Zn-containing superoxide dismutase of *Rhodobacter sphaeroides*. FEMS Microbiol Lett 234:261–267.

S13. Wayne LG, Diaz GA. 1986. A double staining method for differentiating between two classes of mycobacterial catalase in polyacrylamide electrophoresis gels. Anal Biochem 157:89–92.
